# Immunotherapy in breast cancer: current landscape and emerging trends

**DOI:** 10.1186/s40164-025-00667-y

**Published:** 2025-05-22

**Authors:** Ran Ran, Xi Chen, Jin Yang, Binghe Xu

**Affiliations:** 1 Cancer Center, The First Affiliated Hospital of Xi’an Jiaotong University, Xi’an, China; 2 Precision Medicine Center, The First Affiliated Hospital of Xi’an Jiaotong University, Xi’an, China; 3 National Cancer Center, Cancer Hospital, Chinese Academy of Medical Sciences and Peking Union Medical College, Beijing, China

**Keywords:** Breast cancer, Immunotherapy, Immune-checkpoint inhibitors, Combination therapy, Clinical trials

## Abstract

Breast cancer remains one of the most prevalent malignancies worldwide, underscoring an urgent need for innovative therapeutic strategies. Immunotherapy has emerged as a transformative frontier in this context. In triple-negative breast cancer (TNBC), the combination of immunotherapy based on PD-1/PD-L1 immune checkpoint inhibitors (ICIs) with chemotherapy has proven efficacious in both early and advanced clinical trials. These encouraging results have led to the approval of ICIs for TNBC, opening up new therapeutic avenues for challenging-to-treat patient populations. Furthermore, a multitude of ongoing trials are actively investigating the efficacy of immunotherapy-based combinations, including ICIs in conjunction with chemotherapy, targeted therapy and radiation therapy, as well as other novel strategies such as bispecific antibodies, CAR-T cells and cancer vaccines across all breast cancer subtypes, including HR-positive/HER2-negative and HER2-positive disease. This review provides a comprehensive overview of current immunotherapeutic approaches in breast cancer, highlighting pivotal findings from recent clinical trials and the potential impact of these advancements on patient outcomes.

## Introduction

Breast cancer stands out as one of the most common malignant tumor globally, with multiple subtypes and complex immune evasion mechanisms [1, 2]. Since cancer immunotherapy was named one of the top ten scientific breakthroughs of the year by *Science* in 2013, the development of immunotherapy has witnessed groundbreaking advancements and paradigm shifts in the treatment of diverse malignancies. Historically, breast cancer has been considered an immunologically ‘cold’ tumor with reduced levels of T-cell infiltration and lower mutational load compared to other ‘hot’ solid tumors such as non-small cell lung cancer (NSCLC) and melanoma [3]. These distinct immunological characteristics initially limited the exploration of immunotherapy in breast cancer. In recent years, evidence is mounting that the immune system plays a vital role in breast tumor progression, treatment sensitivity and resistance, while immunotherapy has gradually provided new avenues, particularly for refractory subtypes like triple-negative breast cancer (TNBC). As the most immunogenic subtype, TNBC exhibits high programmed death ligand 1 (PD-L1) expression, abundant tumor-infiltrating lymphocytes (TILs) [4], as well as genomic instability and a high mutation rate, which may lead to neoantigen production and increased immunogenicity [5, 6]. The programmed cell death 1 (PD-1)/PD-L1 pathway is a major immune escape mechanism in tumors, and blocking the PD-1/PD-L1 axis has emerged as a promising therapeutic target to enhance antitumor immunity [7]. Several randomized clinical trials have demonstrated that immune checkpoint inhibitors (ICIs) combined with chemotherapy has yielded encouraging results in both early and advanced TNBC. However, challenges persist in patient selection, resistance mechanisms, durability of response, and management of immune-related adverse events (AEs) [8]. Ongoing research aimed at integrating predictive biomarkers, developing more effective combination therapies, and overcoming the immunosuppressive tumor microenvironment (TME) is critical to realizing the full therapeutic potential of breast cancer immunotherapy. Currently, numerous clinical trials are exploring the potential of expanding ICI therapy beyond TNBC to other subtypes. Meanwhile, innovative approaches such as bispecific antibodies (BsAbs), adoptive cell therapies (ACTs), and breast cancer vaccines are emerging as active areas of research.

This review outlines the recent advances in immunotherapy across all breast cancer subtypes and provides a comprehensive overview of both reported and investigational ICI-based combinations and novel immunotherapeutic strategies.

## Immunotherapy for metastatic breast cancer

### ICI for metastatic TNBC

**Fig. 1:**
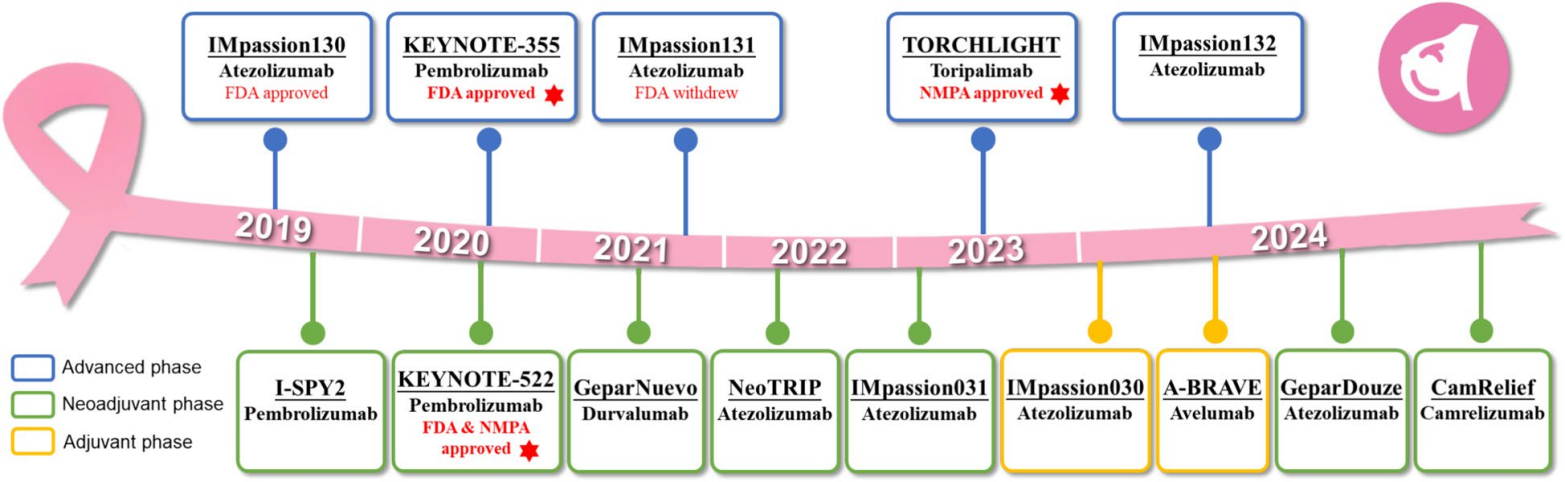
Historic landmark clinical trials of ICI combined with chemotherapy for TNBC. Blue boxes represent clinical trials in advanced settings, green boxes represent clinical trials in neoadjuvant settings, and yellow boxes represent clinical trials in adjuvant settings. Abbreviations: *CT* chemotherapy, *FDA* Food and Drug Administration, *NMPA* National Medical Products Administration, *TNBC* triple-negative breast cancer

A pivotal development in immunotherapy for breast cancer has been the modulation of immune checkpoints, particularly the PD-1/PD-L1 pathway. Immune checkpoint inhibitors (ICIs), such as pembrolizumab, atezolizumab and toripalimab, have shown significant efficacy in clinical trials, especially in TNBC, a subtype characterized by its aggressive nature and lack of targeted therapies. These inhibitors have demonstrated the ability to enhance anti-tumor immunity by blocking inhibitory signals that suppress T-cell activation, leading to improved progression-free survival (PFS) and overall survival (OS) in patients with advanced or metastatic disease. Figure 1 illustrates the pivotal randomized controlled trials of ICI combined with chemotherapy in both early and advanced TNBC in a timeline format.

### ICI monotherapy

Early-phase trials evaluated PD-1/PD-L1 ICIs as monotherapy in patients with heavily pretreated metastatic TNBC (mTNBC). Both pembrolizumab (KEYNOTE-012/086) and atezolizumab demonstrated antitumor efficacy and manageable safety profile, with higher response rates observed in first-line treatment or PD-L1-positive (PD-L1+) disease [9–12]. The phase Ib JAVELIN study suggested that single-agent avelumab showed an acceptable safety profile and clinical activity in mTNBC patients [13]. In a phase I trial (NCT02838823), toripalimab monotherapy displayed a favorable safety profile and moderate response in mTNBC patients after multi-line systemic therapy [14]. However, in the phase III KEYNOTE-119 study, pembrolizumab monotherapy failed to achieve a meaningful OS improvement compared with chemotherapy in the second- or third-line mTNBC, including PD-L1 combined positive score (CPS) ≥1 or ≥10 subgroups. Notably, in the pembrolizumab group, higher PD-L1 expression was associated with a longer median OS, the greatest benefit was observed in patients with PD-L1 CPS ≥20 [15]. These findings suggested that ICI monotherapy may provide limited survival benefits, except in previously untreated mTNBC patients with high PD-L1 expression.

### ICI combined with chemotherapy

Given the suboptimal responses towards ICI monotherapy and the intriguing immunomodulatory properties exhibited by conventional chemotherapeutic agents [16], a number of phase III clinical studies have evaluated the synergistic potential of combining ICIs with chemotherapy for mTNBC and have made dramatic breakthroughs. Landmark clinical trials of ICI plus chemotherapy for mTNBC are summarized in Table 1.

**Table 1:** Phase III randomized clinical trials of ICI plus chemotherapy for mTNBC

Trial name	Phase	Setting	Immunotherapy and control arms	PFS (ICI vs placebo)	OS (ICI vs placebo)	TRAE Grade > 3 (ICI vs placebo)	REF
IMpassion130	III	Previously untreated mTNBC	Immunotherapy (n = 451): Atezo + nab-paclitaxel Control (n = 451): Placebo + nab-paclitaxel	**PD-L1 + (IC ≥ 1%)**^a^ **7.5 vs 5.0 months** **HR, 0.62** **(95% CI, 0.49 to 0.78; *P* < 0.001)**	**PD-L1 + (IC ≥ 1%)**^a^ **25.4 vs 17.9 months** **HR, 0.67** **(95% CI, 0.53 to 0.86)**	48.7% vs 42.2%	[17, 19]
				**ITT** **7.2 vs 5.5 months** **HR, 0.80** **(95% CI, 0.69 to 0.92; *P* = 0.002)**	ITT 21.0 vs 18.7 months HR, 0.87 (95% CI, 0.75 to 1.02; *P* = 0.077)		
IMpassion131	III	Previously untreated mTNBC	Immunotherapy (n = 431): Atezo + paclitaxel Control (n = 220): Placebo + paclitaxel	PD-L1 + (IC ≥ 1%)^a^ 6.0 vs 5.7 months HR, 0.82 (95%CI, 0.60 to 1.12; *P* = 0.20)	PD-L1 + (IC ≥ 1%)^a^ 28.3 vs 22.1 months HR, 1.11 (95% CI, 0.76 to 1.64)	11% vs 5%	[20]
				ITT 5.6 vs 5.7 months HR, 0.86 (95% CI, 0.70 ~ 1.05)	ITT 19.2 vs 22.8 months HR, 1.11 (95% CI, 0.76 to 1.64)		
KEYNOTE-355	III	Previously untreated mTNBC	Immunotherapy (n = 566): Pembro + investigator’s choice of chemotherapy (nab-paclitaxel, paclitaxel, or gemcitabine-carboplatin) Control (n = 281): Placebo + investigator’s choice of chemotherapy (nab-paclitaxel, paclitaxel, or gemcitabine-carboplatin)	**PD-L1 + (CPS ≥ 10)**^b^ **9.7 vs 5.6 months; HR, 0.65** **(95% CI, 0.49 to 0.86; *P* = 0.0012)**	**PD-L1 + (CPS ≥ 10)**^b^ **23.0 vs 16.1 months** **HR, 0.73** **(95% CI, 0.55 to 0.95; *P* = 0.0185)**	68.1% vs 66.9%	[24]
				PD-L1 + (CPS ≥ 1) 7.6 vs 5.6 months HR, 0.74 (95% CI, 0.61 to 0.90; *P* = 0.0014)	PD-L1 + (CPS ≥ 1) 17.6 vs 16.0 months HR, 0.86 (95% CI, 0.72 to 1.04; *P* = 0.1125)		
				ITT 7.5 vs 5.6 months HR, 0.82 (95% CI, 0.69 to 0.97)	ITT 17.2 vs 15.5 months HR, 0.89 (95% CI, 0.76 to 1.05)		
TORCHLIGHT	III	Previously untreated mTNBC	Immunotherapy (n = 353): Toripa + nab-paclitaxel Control (n = 178): Placebo + nab-paclitaxel	**PD-L1 + (CPS ≥ 1)** **8.4 vs 5.6 months** **HR, 0.65 (95% CI, 0.470 to 0.906; *P* = 0.0102)**	PD-L1 + (CPS ≥ 1) 32.8 vs 19.5 months HR, 0.62 (95% CI, 0.414 to 0.914; *P* = 0.0148)	56.4% vs 54.3%	[25]
				ITT 8.4 vs 6.9 months HR, 0.77 (95% CI, 0.602 to 0.994; *P* = 0.0445)	ITT 33.1 vs 23.5 months HR, 0.69 (95% CI, 0.513 to 0.932; *P* = 0.0145)		
IMpassion132	III	mTNBC relapsing < 12 months	Immunotherapy (n = 188): Atezo + carboplatin/ gemcitabine or capecitabine Control (n = 192): Placebo + carboplatin/gemcitabine or capecitabine	PD-L1 + (IC ≥ 1%)^a^ 4.2 vs 3.6 months HR, 0.84 (95% CI, 0.67 to 1.06)	PD-L1 + (IC ≥ 1%)^a^ 12.1 vs 11.2 months HR, 0.93 (95% CI, 0.73 to 1.20, *P* = 0.59)	62% vs 65%	[23]
				ITT 3.7 vs 3.6 months HR, 0.96 (95% CI, 0.78 to 1.19)	ITT 10.4 vs 9.8 months HR, 0.94 (95% CI, 0.76 to 1.18)		

Bolded items represent statistically significant results

Abbreviations: *Atezo* atezolizumab, *CI* confidence interval, *CPS* combined positive score, *HR* hazard ratio, *ICI* immune checkpoint inhibitor, *IHC* immunohistochemistry, *TRAE* treatment‐related adverse event, *ITT* intention-to-treat population, *mTNBC* metastatic triple-negative breast cancer, *OS* overall survival, *PD-L1* programmed death-ligand 1, *PFS* progression-free survival, *Pembro* pembrolizumab, *REF* reference, *Toripa* Toripalimab

^a^For atezolizumab, PD-L1 + is defined by ≥ 1% expression on immune cells as determined by Ventana SP142 IHC assay

^b^For pembrolizumab, PD-L1+ is defined by CPS ≥10 as determined by Dako 22C3 IHC assay. CPS is calculated by adding all PD-L1-positive cells (lymphocytes, macrophages, and tumors) divided by the total number of viable tumor cells

Atezolizumab is the first PD-L1 monoclonal antibody to be demonstrated by the IMpassion130 study that its combined with nab-paclitaxel significantly improved progression-free survival (PFS) in both the PD-L1+ and intention-to-treat (ITT) populations compared to nab-paclitaxel alone [17]. In light of this positive result, in March 2019, atezolizumab plus nab-paclitaxel received accelerated approval from the Food and Drug Administration (FDA) for the first-line treatment of patients with mTNBC whose tumors exhibit PD-L1 expression of ≥1% immune cells (ICs) [18, 19]. However, in the subsequent confirmatory IMpassion131 trial, the combination of paclitaxel with atezolizumab failed to significantly improve PFS and OS for previously untreated mTNBC [20]. Consequently, in August 2021, Roche voluntarily withdrew the indication for the use of atezolizumab plus nab-paclitaxel in patients with PD-L1+ mTNBC [21]. Zhang et al. performed single-cell RNA sequencing on 22 mTNBC patients receiving paclitaxel with or without atezolizumab, suggesting that paclitaxel selectively impaired atezolizumab-induced expansion of responsive ICs, such as specific types of T cells and dendritic cells, thereby affecting the efficacy of atezolizumab combination therapy [22]. Unfortunately, for PD-L1+ mTNBC patients with early relapses, IMpassion132 trial again failed to demonstrate that the addition of atezolizumab to chemotherapy can improve OS [23].

Pembrolizumab is currently the only FDA-approved PD-1 monoclonal antibody to be administered with chemotherapy for patients with PD-L1+ (CPS ≥10) mTNBC in the first-line setting. This was based on significantly prolonged OS and PFS in the KEYNOTE-355 trial, which led to the rapid approval of this combination regimen in Europe and worldwide [24].

Toripalimab, another promising anti-PD-1 antibody, significantly improved PFS (with extended OS trend) in patients with previously untreated PD-L1+ (CPS ≥1) mTNBC when combined with nab-paclitaxel in the TORCHLIGHT study [25]. Given these positive outcomes, in May 2023, China’s National Medical Products Administration (NMPA) accepted a marketing application for toripalimab combined with nab-paclitaxel in patients with previously untreated PD-L1+ (CPS ≥1) mTNBC.

Metronomic chemotherapy is administered more frequently, at lower doses, and has a more potent immunostimulatory effect than conventional chemotherapy [26–29]. The phase II NCT04389073 trial investigated toripalimab combined with metronomic chemotherapy versus conventional chemotherapy in patients with HER2-negative (HER2-) metastatic breast cancer (MBC) using Bayesian adaptive randomization and efficacy monitoring. A total of 97 patients were randomly assigned to five different treatment arms: metronomic vinorelbine (NVB) monotherapy, NVB + anti-PD-1 toripalimab, anti-angiogenic bevacizumab + NVB + toripalimab, conventional cisplatin + NVB + toripalimab, or metronomic cyclophosphamide + capecitabine + NVB + toripalimab (the VEX cohort). Final results suggested that the VEX cohort displayed the best disease control rate (DCR) and longest PFS in the total population (DCR, 69.7%; PFS, 6.6 months) and especially in the TNBC subgroup (DCR, 74.1%; PFS, 9.8 months). These findings demonstrated that toripalimab plus metronomic VEX chemotherapy can achieve obvious and durable synergistic effects, opening up new possibilities for selecting suitable chemotherapeutic backbones for immunotherapy in breast cancer [30].

### Immunotherapy for metastatic HR-positive/HER2-negative breast cancer

Despite the fact that HR-positive/HER2-negative (HR+/HER2-) breast cancers typically exhibit lower TILs and PD-L1 expression, a minority of these patients still have clinical responses to immunotherapy. Clinical trials of ICI-based combinations in HR+/HER2- MBC are summarized in Table 2. KEYNOTE-028 and JAVELIN showed slight efficacy of single-agent ICI (pembrolizumab and avelumab) in patients with HR+/HER2- MBC [13, 31]. In the context of exploring ICI coupled with chemotherapy, KELLY trial demonstrated encouraging anti-tumor activity of pembrolizumab plus eribulin in patients with heavily pretreated HR+/HER2- MBC [32]. However, NCT03051659 study showed no significant difference between eribulin plus pembrolizumab versus eribulin alone in terms of PFS and objective response rate (ORR) [33]. The ongoing phase III KEYNOTE-B49 study is evaluating the addition of pembrolizumab to chemotherapy in patients with PD-L1+ (CPS ≥1) and HR+/HER2- MBC after progression on prior endocrine therapy [34].

**Table 2:** Clinical trials of ICI-based combination therapy in HR + /HER2- and HER2 + MBC

Disease type	Trial name (sample size)	Design	ICI	Intervention	Results	REF
HR + /HER2–	KEYNOTE-028 (n = 25 HR + and PD-L1 + CPS ≥ 1)	Single-arm phase Ib	Pembro	Pembro monotherapy	ORR: 12.0%; CBR: 20%; DoR: 12 months	[31]
	JAVELIN (n = 72 HR +)	Single-arm phase Ib	Avelumab	Avelumab monotherapy	ORR: 2.8%	[13]
	KELLY (n = 44)	Single-arm phase II	Pembro	Pembro + eribulin	CBR: 56.8%; ORR: 40.9%; PFS: 6.0 months; 1-year OS: 59.1%	[32]
	NCT03051659 (n = 88)	Randomized phase II	Pembro	Pembro + eribulin vs eribulin	PFS: 4.1 vs 4.2 months; *P* = 0.33 ORR: 27% vs 34%; *P* = 0.49	[33]
	NCT02778685 (n = 23)	Single-arm phase I/II	Pembro	Pembro + palbociclib + LET	CR: 31%; PR: 25%; SD: 31% PFS: 25.2 months; OS: 36.9 months	[38]
	PACE (n = 220)	Randomized Phase II	Avelumab	FUL vs FUL + palbociclib vs FUL + palbociclib + avelumab	PFS: 4.8 vs 4.6 vs 8.1 months ORR: 7.3% vs 9.0% vs 13.0% CBR: 29.1% vs 32.4% vs 35.2% OS: 27.5 vs 24.6 vs 42.5 months	[39]
	NCT02779751 (n = 54)	Single-arm phase Ib	Pembro	Pembro + abemaciclib + anastrozole vs Pembro + abemaciclib	Overall response rate: 23.1% vs 28.6% DCR: 84.6% vs 82.1% PFS: immature vs 8.9 months OS: immature vs 26.3 months (Benefit/risk analysis did not support further evaluation of this combination in HR + /HER2– MBC.)	[40]
	NEWFLAME (n = 17)	Nonrandomized phase II	Nivo	Nivo + abemaciclib + FUL vs Nivo + abemaciclib + LET	ORR: 54.5% vs 20% DCR: 90.9% vs 80.0% (The study was discontinued prematurely for enrollment and combination therapy due to safety concerns. AEs ≥ Grade 3: 91.7% vs 100%; irAEs ≥ Grade 3: 66.7% vs 60.0%; SAEs: 50.0% vs 60.0%)	[41]
	CheckMate 7A8 (n = 21)	Single-arm phase Ib/II	Nivo	Nivo + palbociclib + anastrozole	A high incidence of grade 3/4 hepatotoxicity and treatment discontinuation caused the study to be terminated early	[42]
	NCT03294694	Single-arm phase I	Sparta	Sparta + ribociclib ± FUL	ORR: 13.7% (Cohort B, with FUL) (Elevated hepatotoxicity led to early study termination. Grade 3 AEs: 46.7%; Grade 4 AEs: 40.0%)	[43]
	SACI-IO HR + (n = 110)	Randomized phase II	Pembro	Pembro + SG vs SG	PFS: 8.1 vs 6.2 months; HR, 0.81; *P* = 0.37 OS: 18.5 vs 18.0 months; HR, 0.65; *P* = 0.21 ORR: 21.2% vs 17.3% PD-L1 + (CPS ≥ 1) HR + /HER2– MBC: PFS: 11.1 vs 6.7 months, HR, 0.62; *P* = 0.23; OS: 18.5 vs 12.5 months, HR, 0.61; *P* = 0.42	[46]
HER2 +	PANACEA (n = 55)	Phase Ib/II parallel cohort	Pembro	Pembro + trastuzumab	PD-L1 + vs PD-L1–: ORR: 15% vs 0% PFS: 2.7 vs 2.5 months 12-month PFS rate: 12% vs 0% 12-month OS rate: 65% vs 12%	[48]
	NRG BR004 (n = 200)	Randomized phase III	Atezo	Atezo** + **THP vs THP	2-year PFS rate: 54.0% vs 45.6%, *P* = 0.12 3-year OS rate: 86.4% vs 81.7%, *P* = 0.53 (The study was terminated prematurely due to safety concerns)	[49]
	KATE2 (n = 202)	Randomized phase II	Atezo	Atezo or placebo + T-DM1	ITT: PFS: 8.2 vs 6.8 months; HR, 0.82; *P* = 0.33 PD-L1 + : PFS: 8.5 vs 4.1 months; HR, 0.60; *P* = 0.099 PD-L1-: PFS: 6.8 vs 8.2 months; HR, 1.02; *P* = 0.33	[50]
	NCT03523572 (n = 48)	Single-arm phase Ib	Nivo	Nivo + T-DXd	HER2 + vs HER2-low: ORR: 59.4% vs 37.5% DCR: 90.6% vs 75.0% PFS: 8.6 vs 6.3 months	[54]

Abbreviations: *Atezo* atezolizumab, *CBR* clinical benefit rate, *CR* complete response, *DCR* disease control rate, *DoR* duration of response, *FUL* fulvestrant, *HR* hazard ratio, *ICI* immune checkpoint inhibitor, *irAEs, IrAE* immune-related adverse events, *ITT* intention-to-treat population, *LET* letrozole, *MBC* metastatic breast cancer, *Nivo* nivolumab, *ORR* objective response rate, *OS* overall survival, *Pembro* pembrolizumab, *PD-L1* programmed death-ligand 1, *PFS* progression-free survival, *PR* partial response, *REF* reference, *SAEs* severe adverse events, *SD* stable disease, *Sparta* spartalizumab, *THP* taxane plus trastuzumab and pertuzumab

Preclinical studies indicated that CDK4/6 inhibitors increased tumor immunogenicity [35] and synergized PD-1 blockade to enhance antitumor efficacy [36, 37]. In the NCT02778685 trial, the triple regimen of palbociclib, pembrolizumab and letrozole as first-line therapy reached a complete response rate of 31% in HR+/HER2- MBC patients and was well tolerated [38]. PACE trial suggested the addition of avelumab to palbociclib and fulvestrant increased PFS in patients with HR+/HER2- MBC who had progressed on prior treatment with CDK4/6 inhibitor and aromatase inhibitor [39]. Nevertheless, several trials evaluating the combinations of ICI plus CDK4/6 inhibitors and aromatase inhibitor were prematurely terminated due to safety concerns including unacceptable rates of hepatotoxicity and interstitial lung disease or pneumonitis, which did not support further studies [40–43]. Poor tolerability of ICI combined with CDK4/6 inhibitor remains a hurdle for clinical trials.

Sacituzumab govitecan (SG) is a TROP2-targeted drug conjugate (ADC) with a topoisomerase I inhibitor payload (SN-38), approved by the FDA for patients with heavily pretreated mTNBC and HR+/HER2- MBC [44, 45]. Preliminary analysis from the SACI-IO HR+ trial revealed that pembrolizumab plus SG achieved a numerically non-statistical improvement in PFS for HR+/HER2- MBC patients. Among PD-L1+ patients, pembrolizumab plus SG resulted in a 4.4-month increase in PFS and a 6-month increase in OS compared to SG monotherapy. However, there was no statistical difference in PFS and OS between patients stratified by PD-L1 expression (CPS ≥1 vs CPS <1) [46].

### Immunotherapy for metastatic HER2-positive breast cancer

The combination of HER2-targeted antibodies and immunostimulatory antibodies against PD-1 and CD137 demonstrated synergistic preclinical anti-tumor activity, which supported the clinical evaluation of combination therapy with HER2-targeted and PD-(L)1 antibodies [47]. Several clinical trials investigated the combination of ICI and trastuzumab/trastuzumab-based ADCs in HER2-positive (HER2+) MBC, and the reported responses were mainly occurred in PD-L1+ patients (as illustrated in Table 2).

PANACEA trial evaluated the addition of pembrolizumab to trastuzumab in patients with trastuzumab-resistant HER2+ MBC. The results showed that pembrolizumab was more effective in the PD-L1+ (CPS ≥1) group than in the PD-L1-negative (PD-L1–) group. Subgroup analyses showed significantly higher TIL levels in the PD-L1+ tumors than in the PD-L1– tumors [48]. NRG BR004 trial explored the addition of atezolizumab to the THP regimen (taxane-based dual-targeted therapy with trastuzumab and pertuzumab) as first-line treatment in HER2+ MBC. Unfortunately, the results of this study did not meet expectations and terminated prematurely due to higher mortality in the atezolizumab group [49].

Concerning the combination of ICI and ADC, KATE2 study evaluated atezolizumab plus trastuzumab emtansine (T-DM1) versus placebo plus T-DM1 in patients with HER2+ MBC that had progressed on trastuzumab and a taxane. While no clinical benefit was seen in the ITT population, PD-L1+ subgroups showed PFS improvement (though no OS benefit) with increased AEs [50]. The ongoing phase III KATE3 is exploring this combination exclusively in PD-L1+ patients, with both PFS and OS as co-primary endpoints. Trastuzumab deruxtecan (T-DXd), another promising ADC, has demonstrated remarkable antitumor efficacy in HER2+ and HER2-low breast cancer [51–53]. Interim analysis of the NCT03523572 trial suggested that T-DXd plus nivolumab had antitumor activity and an acceptable safety profile in HER2-expressing MBC [54]. Moreover, The combination of T-DXd with durvalumab and paclitaxel is being investigated in the phase Ib/II DESTINY-Breast 07 trial (NCT04538742).

## Immunotherapy for early breast cancer

### ICI for early TNBC

Proven success of ICI therapy in mTNBC has facilitated the shift of clinical studies on ICI plus chemotherapy regimens towards earlier-stage settings. Of note, evidence suggests that the early disease setting, especially in the neoadjuvant setting, are probably the the most opportune period for ICI therapy, during this time, the immune microenvironment is more conducive and host immune system is less compromised prior to the acquisition of multiple immune escape mechanisms [55–57]. Table 3 summarizes the key randomised controlled trials of ICI therapy for early-stage TNBC (eTNBC).

**Table 3:** Phase II/III randomized clinical trials of neoadjuvant/adjuvant ICI therapy for eTNBC

Trial name	Phase	Setting	Immunotherapy and Control arms	pCR rate (95% CI) (ICI vs placebo)	EFS/DFS/OS (ICI vs placebo)	REF
I-SPY2	II	Neoadjuvant	Immunotherapy (n = 69): Pembro + paclitaxel × 4 → AC × 4 → surgery Control (n = 201): paclitaxel × 4 → AC × 4 → surgery	**60% (44% to 75%) vs 22% (13% to 30%)**	Numerically higher but not powered for statistical significance	[59]
GeparNuevo	II	Neoadjuvant	Immunotherapy (n = 88): Durva + nab-paclitaxel × 4 → durva + EC × 4 → surgery Control (n = 86): nab-paclitaxel × 4 → EC × 4 → surgery	ITT 53.4% (42.5% to 61.4%) vs 44.2% (33.5% to 55.3%) (OR, 1.45; *P* = 0.287)	**3-year iDFS: 85.6% vs 77.2% (HR, 0.48; 95% CI, 0.24 to 0.97; *P* = 0.036)** **3-year DDFS: 91.7% vs 78.4% (HR, 0.31; 95% CI, 0.13–0.74; *P* = 0.005)** **3-year OS: 95.2% vs 83.5% (HR, 0.24; 95% CI, 0.08 to 0.72; *P* = 0.006)**	[70, 71]
				**Window cohort** **61% vs 41.4% (OR, 2.22; 95% CI, 1.06 to 4.64; *P* = 0.035)**		
NCI 10013	II	Neoadjuvant	Immunotherapy (n = 45): Atezo + carboplatin + paclitaxel × 4 → surgery → ddAC × 4 Control (n = 22): carboplatin + paclitaxel × 4 → surgery → ddAC × 4	**ITT** **55.6% (40.0% to 70.4%) vs 18.8% (4.0% to 45.6%) (95% CI, 8.5% to 56.6%; *P* = 0.018)**	Not reported	[74]
				**PD-L1 positive** **75.0% vs 0% (*P* = 0.01)**		
				PD-L1 negative 31.6% vs 20% (*P* > 0.99)		
KEYNOTE-522	III	Neoadjuvant + adjuvant	Immunotherapy (n = 784): Pembro + paclitaxel + carboplatin → pembro + AC/EC × 4 → surgery → pembro + chemotherapy × 9 Control (n = 390): paclitaxel + carboplatin + placebo → AC/EC + placebo × 4 → surgery → placebo + chemotherapy × 9	**ITT** **64.8% (59.9% to 69.5%)** **vs 51.2% (44.1% to** **58.3%) (*P* < 0.001)**	**5-year EFS: 81.2% (95% CI, 78.3 to 83.8) vs 72.2% (95% CI, 67.4 to 76.4) (HR, 0.65; 95% CI, 0.51 to 0.83) (*P* < 0.001)** **5-year OS: 86.6% (95% CI, 84.0 to 88.8) vs 81.7% (95% CI, 77.5 to 85.2) (*P* = 0.002)**	[60–62]
				PD-L1 positive 68.9% vs 54.9%		
				PD-L1 negative 45.3% vs 30.3%		
				LN negative 64.9% vs 58.6%		
				LN positive 64.8% vs 44.1%		
NeoTRIPaPD-L1	III	Neoadjuvant	Immunotherapy (n = 138): Atezo + nab-paclitaxel + carboplatin × 8 → surgery → AC/ EC/5 FU + EC × 4 Control (n = 142): nab-paclitaxel + carboplatin × 8 → surgery → AC/EC/5 FU + EC × 4	ITT 48.6% vs 44.4% (32.7% to 49.4%) (OR, 1.18; 95% CI, 0.74 to1.89; *P* = 0.48)	5-year EFS: 70.6% (95% CI, 61.6 to 77.9) vs 74.9% (95% CI, 66.6 to 81.5) (HR, 1.076; 95% CI, 0.670 to 1.731; *P* = 0.76)	[63, 64]
				ICI group PD-L1 positive vs PD-L1 negative: 59.5% vs 33.9% (OR, 2.86; 95% CI, 1.42 to 5.78; *P* = 0.03)		
				Placebo group PD-L1 positive vs PD-L1 negative: 51.9% vs 35.4% (OR, 1.55; 95% CI, 0.8 to 3.0; *P* = 0.18)		
IMpassion031	III	Neoadjuvant + adjuvant	Immunotherapy (n = 168): Atezo + nab-paclitaxel × 12 → atezo + ddAC × 8 → surgery → atezo × 11 Control (n = 165): placebo × 6 + nab-paclitaxel × 12 → placebo + ddAC × 8 → surgery → monitoring	**ITT** **58% (50% to 65%) vs** **41% (34% to 49%) (*P* = 0.0044)**	ITT 2-year EFS: 85% (79% to 90%) vs 80% (74% to 86%) (HR, 0.76; 95% CI, 0.44 to 1.30) 2-year DFS: 87% (82% to 93%) vs 83% (77% to 89%) (HR, 0.76; 95% CI, 0.47 to 1.21) 2-year OS: 95% (91% to 98%) vs 90% (85% to 95%) (HR, 0.56; 95% CI, 0.30 to 1.04)	[65, 66]
				**PD-L1 positive** **69% (57% to 79%) vs** **49% (38% to 61%) (*P* = 0.021)**	PD-L1 positive 2-year EFS: 89% (82% to 96%) vs 80% (71% to 89%) (HR, 0.55; 95% CI, 0.21 to 1.18) 2-year DFS: 91% (85% to 98%) vs 87% (79% to 95%) (HR, 0.57; 95% CI, 0.23 to 1.43) 2-year OS: 95% (89% to 100%) vs 91% (84% to 98%) (HR, 0.71; 95% CI, 0.26 to 1.91)	
IMpassion030	III	Adjuvant	Immunotherapy (n = 1101): surgery → atezo + paclitaxel × 12 → atezo + ddAC/EC × 4 → atezo to complete 1 year Control (n = 1098): surgery → paclitaxel × 12 → ddAC/EC × 4	None	ITT iDFS events: 12.8% vs 11.4 (HR, 1.11; 95% CI, 0.87 to 1.42; *P* = 0.38) OS events: 6.5% vs 5.3% (HR, 1.23; 95% CI, 0.87 to 1.73)	[77]
					PD-L1 positive iDFS events: 10.6% vs 10.4 (HR, 1.00, 95% CI, 0.73 to 1.35)	
A-BRAVE	III	Adjuvant	Immunotherapy (n = 238): surgery → avelumab to complete 1 year Control (n = 239): surgery → observation	None	3-year DFS: 66.9% vs 60.7% (HR, 0.80; 95% CI, 0.58 to 0.97; *P* = 0.170) **3-year OS: 84.8% vs 76.3% (HR, 0.66; 95% CI, 0.45 to 0.97; *P* = 0.035)** **3-year DDFS: 75.4% vs 67.9% (HR, 0.70; 95% CI, 0.50 to 0.96; *P* = 0.0277)**	[79]
GeparDouze	III	Neoadjuvant	Immunotherapy (n = 773): Atezo + wP + carboplatin × 8 → atezo + AC/EC × 4 → surgery → atezo to complete 1 year Control (n = 777): placebo + wP + carboplatin × 8 → placebo + AC/EC × 4 → surgery → placebo	ITT 63.3% (53.5% to 60.5%) vs 57.0% (59.9% to 66.7%) (*P* = 0.091)	4-year EFS: 85.2% (82.4% to 87.7%) vs 81.9% (78.9%, 84.6%) (HR, 0.8; 95% CI, 0.62 to 1.03; *P* = 0.08) 4-year OS: 90.2% (87.7% to 92.3%) vs 89.5% (86.9% to 91.5%) (HR, 0.86; 95% CI, 0.62 to 1.19)	[67]
CamRelief	III	Neoadjuvant	Immunotherapy (n = 222): Camre + nab-paclitaxel + carboplatin × 8 → camre + EC × 4 → surgery → camre to complete 1 year Control (n = 219): placebo + nab-paclitaxel + carboplatin × 8 → placebo + EC × 4 → surgery → standard care	**ITT** **56.8% (50.0% to 63.4%) vs 44.7% (38.0% to 51.6%) (*P* = 0.004)**	Not reported	[69]

Bolded items represent statistically significant results

Abbreviations: *AC* doxorubicin/cyclophosphamide, *Atezo* atezolizumab, *Camre* camrelizumab, *CI* confidence interval, *DDFS* distant disease-free survival, *ddAC/EC* dose dense anthracycline/epirubicin and cyclophosphamide, *DFS* disease-free survival, *Durva* durvalumab, *EC* epirubicin/cyclophosphamide, *EFS* event-free survival, *eTNBC* early triple-negative breast cancer, *HR* hazard ratio, *ICI* immune checkpoint inhibitor, *iDFS* Invasive disease-free survival, *ITT* intent-to-treat, *LN* lymph node, *OR* odds ratio, *pCR* pathologic complete response, *PD-L1* programmed death-ligand 1, *Pembro* pembrolizumab, *REF* reference, *SAE* serious adverse event, *wP* weekly paclitaxel

### Neoadjuvant setting

In succession, three studies confirmed the key position of pembrolizumab in eTNBC. The phase Ib KEYNOTE-173 trial explored neoadjuvant pembrolizumab plus chemotherapy in high-risk eTNBC patients, demonstrating a 60% pathologic complete response (pCR) rate across all cohorts, with efficacy positively correlated with PD-L1 expression and stromal TIL levels. In addition, 1-year EFS and OS rates reached 80–100% across all arms [58]. I-SPY2 trial is a phase II adaptive platform designed to evaluate multiple new agents combined with standard therapy, with pCR as the primary endpoint. In the TNBC cohort, adding pembrolizumab to the standard neoadjuvant chemotherapy (NACT) substantially increased the pCR rate versus NACT alone (60% vs 20%) [59]. Subsequently, the phase III KEYNOTE-522 trial demonstrated significant improvements in pCR and EFS with the addition of pembrolizumab to neoadjuvant paclitaxel and carboplatin [60, 61]. Furthermore, the EFS benefit was concordant across subgroups irrespective of PD-L1 status [61]. Based on these results, in July 2021, the FDA approved pembrolizumab combined with chemotherapy as neoadjuvant therapy for high-risk eTNBC patients with continued pembrolizumab monotherapy as adjuvant therapy after surgery. Recently published final OS results showed that pembrolizumab-chemotherapy regimen significantly prolonged OS compared to chemotherapy alone [62].

Three phase III trials have published paradoxical findings on the assessment of atezolizumab in eTNBC. NeoTRIP trial showed that atezolizumab combined with nab-paclitaxel and carboplatin failed to significantly increase the pCR rate and 5-year EFS, however, patients with higher PD-L1 expression had a markedly superior pCR rate in the atezolizumab group [63, 64]. Nevertheless, IMpassion031 trial indicated that the addition of atezolizumab to a chemotherapy regimen based on nab-paclitaxel and anthracyclines for eTNBC provided a statistically significant pCR benefit and numerically improved EFS, DFS and OS. In exploratory analyses, pCR was highly predictive for long-term outcome, while positive ctDNA at/after surgery was linked to poor DFS and OS [65, 66]. GeparDouze trial recently reported at the San Antonio Breast Cancer Symposium (SABCS) in 2024, suggested that the addition of atezolizumab to NACT followed by adjuvant atezolizumab increased pCR from 57 to 63%, but without EFS benefit [67].

Camrelizumab, an anti-PD-1 antibody, in combination with chemotherapy has presented excellent efficacy in eTNBC. In a single-arm phase II trial, camrelizumab plus nab-paclitaxel and epirubicin as neoadjuvant therapy reported a pCR rate of 64.1% and an ORR of 89.7% [68]. The phase III CamRelief trial demonstrated that the addition of camrelizumab to NACT (epirubicin, cyclophosphamide, nab-paclitaxel, and carboplatin) significantly improved pCR rate [69].

Additionally, the other two types of ICIs have showed promising results in phase II clinical trials for eTNBC. In the GeparNUEVO study, the addition of durvalumab to neoadjuvant nab-paclitaxel yielded a modest pCR increase and the durvalumab effect was only observed in the window cohort, in which patients received durvalumab or placebo for two weeks before starting nab-paclitaxel. Notably, secondary endpoints indicated that durvalumab significantly improved long-term survival outcomes including invasive disease-free survival (iDFS), distant disease-free survival (DDFS) and OS, despite the lack of an adjuvant ICI phase. Furthermore, the clinical benefits remained consistent in the duvarizumab group independent of pCR [70, 71]. The single-arm NeoTENNIS study evaluated 12 weeks of toripalimab plus nab-paclitaxel after anthracycline-based neoadjuvant therapy in eTNBC, achieving an encouraging pCR rate of 55.7%, which warrants further investigation [72].

Optimal chemotherapy partners can contribute to maximizing the benefit of ICIs in eTNBC patients. In the KEYNOTE-522 study, the addition of platinum to paclitaxel sequential anthracycline remains the most effective regimen with the highest pCR rate and impressive OS, and the preferred choice recommended by most guidelines. Several studies have examined the efficacy of neoadjuvant de-escalated regimen without anthracycline in eTNBC. The phase II NeoPACT trial investigated an anthracycline-free neoadjuvant regimen of pembrolizumab plus carboplatin and docetaxel, reporting an encouraging pCR rate of 58% and a 3-year EFS of 86%, with 98% in pCR group and 68% in non-pCR group [73]. NCI 10013 trial demonstrated that the addition of atezolizumab to a non-anthracycline-based neoadjuvant regimen (carboplatin and paclitaxel) significantly increased pCR rate, particularly in PD-L1+ patients [74]. The phase II single-arm cTRIO study reported that neoadjuvant tislelizumab (an anti-PD-1 antibody) with nab-paclitaxel and carboplatin, and then adjuvant tislelizumab achieved a high pCR rate of 56.5%, with higher pCR rates in PD-L1 ≥10% versus ≥1% ICs (81.8% vs 53.6%) [75]. These data suggest that anthracycline-free regimens may provide comparable pCR benefits to anthracycline-based immunochemotherapy. Therefore, it remains controversial whether anthracyclines can be omitted in the neoadjuvant setting. To further address this issue, the ongoing phase III SWOG 2212 trial directly compares the anthracycline-free NeoPACT regimen with the standard KEYNOTE-522 regimen, which may offer more evidence for optimizing chemotherapy backbone selection in neoadjuvant immunotherapy [76].

### Adjuvant setting

ALEXANDRA/IMpassion030 trial was the first phase III study to evaluate adjuvant chemotherapy combined with atezolizumab in eTNBC patients. The final analysis revealed that adding atezolizumab to anthracycline/taxane-based adjuvant chemotherapy did not significantly improve iDFS and OS [77]. In the KEYNOTE-522 study, the immunotherapy arm received pembrolizumab in both neoadjuvant and adjuvant settings, making it unclear whether the DFS benefit stemmed from one or both phases. To assess the role of adjuvant pembrolizumab following neoadjuvant therapy, the phase III OptimICE-pCR trial randomized patients who achieved pCR after NACT plus pembrolizumab 1:1 to either 27 weeks of adjuvant pembrolizumab or observation [78]. This study will provide further guidance on adjuvant treatment selection for pCR patients. A-BRAVE trial compared 1 year of avelumab versus observation for eTNBC patients with residual disease after NACT or at high risk after primary surgery and adjuvant chemotherapy. One year adjuvant avelumab did not significantly improve DFS; nevertheless, the secondary enpoind OS was significanlty improved by avelumab, with the risk of distant metastasis and death reduced by 30% and 34%, respectively [79].

SWOG S1418, an ongoing phase III trial is assessing pembrolizumab as adjuvant therapy for TNBC patients with at least 1cm residual invasive cancer and/or positive lymph node following NACT and surgical intervention [80]. One thousand patients are randomized in 1:1 to receive one year of pembrolizumab or observation with the primary endpoint of iDFS and the secondary endpoints of OS and distant relapse-free survival. Although substantial progress has been made with ICI-based immunotherapy in the treatment of eTNBC, it is critical to perform further studies, particularly on the duration of immunotherapy, optimum chemotherapy partners, and predictive biomarkers of response.

### Immunotherapy for early HR + /HER2- breast cancer

HR+/HER2– breast cancer is generally correlated with a favourable prognosis. However, certain patient subgroups, such as those in the premenopausal phase or with high-grade disease, extensive nodal involvement, or low ER expression, exhibit poorer outcomes. A growing body of research suggested that a subset of high-risk patients with HR+/HER2– early breast cancer (EBC) may benefit from ICI therapy (as illustrated in Table 4). In the HR+/HER2– cohort of the I-SPY2 trial, addition of pembrolizumab to NACT led to a meaningful increase in the pCR rate from 13 to 30% [59]. The GIADA trial recruited 43 premenopausal women with stage II-IIIA HR+/HER2- EBC (Ki67 ≥ 20% and/or grade 3) to treated with 3 cycles of EC and then 8 cycles of 2-week nivolumab, with a pCR rate of 16.3% [81].

**Table 4:** Clinical trials of ICI-based combination therapy in HR + /HER2 + and HER2 + EBC

Disease type	Trial name (sample size)	Design	ICI	Intervention	Adjuvant therapy	pCR rate (ICI vs placebo if RCT)	REF
HR + /HER2–	I-SPY2 (n = 40 HR +)	Randomized phase II	Pembro	paclitaxel → AC	physician’s choice	**30% vs 13%**	[59]
	GIADA (n = 43)	Single-arm phase II	Nivo	EC × 3, OFS + exemestane	OFS + exemestane	16.3%	[81]
	KEYNOTE-756 (n = 1278)	Randomized phase III	Pembro	paclitaxel → AC	Pembro × 9 + ET	**24.3% vs 15.6%;** ***P* = 0.00005**	[82]
	Checkmate 7FL (n = 830)	Randomized phase III	Nivo	paclitaxel → AC	Nivo × 7 + ET	**ITT: 24.5% vs 13.8%;** ***P* = 0.0021** CPS ≥ 1: 40.4% vs 23.8% CPS ≥ 10: 65.7% vs 33.3% CPS ≥ 20: 78.9% vs 26.7%	[84, 85]
	FINEST (n = 27 Group C)	Single-arm phase II	Adebre	Dalpiciclib + letrozole ± OFS	NA	pCR: 0% ORR: 81.5%	[86]
HER2 +	Neo-PATH (n = 67)	Single-arm phase II	Atezo	HP + docetaxel	pCR: HP + Atezo × 12 non-pCR: Atezo + T-DM1 × 14	ITT: 61% HR- vs HR + : 77% vs 44% PD-L1 + vs PD-L1-: 100% vs 53%	[91]
	IMpassion050 (n = 454)	Randomized phase III	Atezo	ddAC → paclitaxel + HP	pCR: Atezo/placebo + HP non-pCR: Atezo/placebo + T-DM1	ITT: 62.4% vs 62.7% PD-L1 + : 64.2% vs 72.5%	[92]
	APTneo (n = 661)	Randomized phase III	Atezo	Arm A: HPCT Arm B1: AC + atezo → HPCT + atezo Arm B2: AC → HPCT + atezo	HER2 directed therapies ± atezolizumab until completion of 1 year	B vs A: 57.8% vs 52.0%; *P* = 0.091 B1 vs B2: 61.9% vs 53.6%; *P* = 0.089 **B1 vs A: 61.9% vs 52.0%; *P* = 0.022**	[93]
	Keyriched-1 (n = 48)	Single-arm phase II	Pembro	HP	HP	ITT: 46% HR- vs HR + : 58.5% vs 38.5%	[94]

Bolded items represent statistically significant results

Abbreviations: *Adebre* adebrelimab, *Atezo* atezolizumab, *CI* confidence interval, *CPS* combined positive score, *dose* dense doxorubicin and cyclophosphamide, *HP* trastuzumab and pertuzumab, *HPCT* trastuzumab and pertuzumab plus carboplatin with paclitaxel, *HR* hazard ratio, *ICI* Immune checkpoint inhibitor, *IHC* immunohistochemistry, *ITT* intention-to-treat population, *mTNBC* metastatic triple-negative breast cancer, *NA* not applicable, *Nivo* nivolumab, *ORR* objective response rate, *OS* overall survival, *Pembro* pembrolizumab, *PD-L1* programmed death-ligand 1, *PFS* progression-free survival, *RCT* randomized controlled trial, *REF* reference

Recent data from several randomized phase III trials and real-world study have showed benefits of adding neoadjuvant immunotherapy to chemotherapy in HR+/HER2- EBC patients. KEYNOTE-756 trial demonstrated that pembrolizumab plus chemotherapy as neoadjuvant treatment followed by pembrolizumab plus endocrine as adjuvant treatment significantly improved pCR compared to placebo. This benefit was particularly pronounced in subgroups with high PD-L1 expression and ER-low (1–9%) disease [82]. PROMENADE, a real-world French cohort from 12 cancer centers (n=114), suggested that ER/PR-low (1–9%) HER2– EBC patients achieved a 75% pCR rate with the KEYNOTE-522 regimen. This pCR rate was more comparable to that of TNBC treated with the KEYNOTE-522 regimen rather than HR+/HER2– EBC reported in KEYNOTE-756, which indicated that HR-low/HER2– EBC may be regarded as TNBC to maximize pCR rate [83]. CheckMate 7FL investigated nivolumab versus placebo in combination with NACT and adjuvant endocrine therapy in high-risk HR+/HER2- EBC. Nivolumab significantly improved pCR and residual cancer burden (RCB) 0 to 1 rates overall, particularly in PD-L1+ patients. The pCR benefit derived from nivolumab progressively improved with increasing PD-L1 expression [84, 85]. Although promising, nivolumab is not currently approved by the FDA for breast cancer and further studies are necessary to confirm the benefits of this combination therapy.

In the phase II FINEST trial, 27 patients with HR+/HER2- EBC who were insensitive to chemotherapy (<40% tumor regression after 2 cycles of nab-paclitaxel and carboplatin) receive a combination of adebrelimab (a PD-L1 inhibitor) plus dalpiciclib and letrozole, a regimen conversion that did not increase the pCR rate but improved the ORR and reduced Ki-67 levels [86].

### Immunotherapy for early HER2-positive breast cancer

The immune system contributes substantially to the response/resistance of HER2-targeted therapies [87–89]. The addition of ICIs to anti-HER2 treatment has shown synergistic efficacy in preclinical data, and the combination has been examined in several clinical trials (as illustrated in Table 4) [47, 90].

A few studies have reported mixed results for the use of atezolizumab in HER2+ EBC. Neo-PATH trial evaluated atezolizumab combined with trastuzumab-pertuzumab (HP) and docetaxel in the neoadjuvant setting, and achieved a 61% pCR rate in the ITT population, with higher rates in HR- and PD-L1+ subgroups [91]. However, IMpassion050 found no pCR benefit with atezolizumab added to dose-dense doxorubicin/cyclophosphamide (AC)-paclitaxel and HP, either in the ITT or PD-L1+ populations [92]. APTneo trial showed that the addition of atezolizumab to NACT and HP resulted in a non-significant 5.8% increase of pCR rate. However, exploratory analysis revealed significantly improved pCR when atezolizumab was added to AC followed by HP plus carboplatin and paclitaxel (HPCT), suggesting either a direct anthracycline’s effect or a mechanistic enhancement of AC with atezolizumab [93].

Keyriched-1 trial evaluated a NACT-free 12-week de-escalation dual anti-HER2 blockade with HP and pembrolizumab in HER2+ EBC. In the primary analysis, the pCR rate for this triple therapy was 46%, with a higher pCR rate in HR- tumors than in HR+ tumors (58.5% vs 38.5%) [94]. Separately, for HER2+ EBC patients with residual disease after neoadjuvant anti-HER2 therapy plus chemotherapy, the phase III ASTEFANIA trial is evaluating the benefit of adding atezolizumab to adjuvant T-DM1 [95]. Although most results of ICI combined with HER2-targeted therapy have been positive, as with HR+/HER2– EBC, clinical trials for HER2+ EBC only show in pCR and response rates benefits rather than efficacy-driven endpoints, and survival-based endpoints are required to confirm the efficacy of ICI in HER2+ EBC.

## Biomarkers for predicting immunotherapy response

Exploration of various potential biomarkers to predict immunotherapy response in breast cancer, such as PD-L1 expression, TILs, microsatellite instability (MSI)/mismatch repair (MMR) defects, and TMB, is currently underway.

### PD-L1 expression

PD-L1 expression in the TME may serve as an immunologic brake on the anti-tumor immune responses [96]. At present, PD-L1 CPS positivity (CPS ≥10) is an FDA-approved definitive biomarker for the administration of pembrolizumab in first-line setting mTNBC. The predictive ability of PD-L1 expression levels for immunotherapeutic response is not consistent across settings. In the advanced setting, the KEYNOTE-355 and IMpassion130 studies suggested that the improved PFS and OS were dependent on PD-L1 expression, and the efficacy of the ICIs increased with elevated levels of PD-L1 expression [17, 24]. Conversely, in early-stage disease, KEYNOTE-522 and IMpassion031 trials revealed an increase in pCR irrespective of PD-L1 status, albeit with a trend towards higher pCR rates in PD-L1-positive patients [60, 65]. Therefore, PD-L1 is not exclusively used as an indicator of the efficacy of immunotherapy, which may be connected with the existence of heterogeneity in PD-L1 levels, as well as the different immune microenvironments between advanced and early-stage patients [97]. To date, there is no standardized PD-L1 assay, immunohistochemistry (IHC) is the primary method for assessing PD-L1 expression in tumor samples, including five commonly used PD-L1 assay kits: SP142, SP263, 22C3, 28-8, and JS311, and their applications in TNBC are listed in Table 5. PD-L1 expression varied among the different assays, with an overlap of positive expression ranging from 63% to 70%, and the four assays, excluding SP142, displayed a high degree of consistency [98, 99]. The FDA has designated four commercial PD-L1 assays as “companion diagnostics”, among which only the 22C3 assay (Dako) is specifically approved for use in breast cancer [100]. Both the 22C3 and JS311 assays employ a CPS scoring system to identify the PD-L1 status of TNBC. If the tumor has CPS ≥10, it was considered PD-L1+ and this subset of patient is eligible for pembrolizumab, whereas in the TORCHLIGHT study, PD-L1+ was defined as JS311 CPS >1 and this subset of patients could benefit from toripalimab. A study using archival FFPE biopsy samples from 103 TNBC patients presented an overall concordance of >85% between 22C3 and JS311 at different CPS cut-off points [25]. Collectively, the present evidence supports the significance of PD-L1 expression in selecting patients with metastatic disease, whereas its implications in early-stage disease remains poorly defined.

**Table 5:** Different PD-L1 immunohistochemistry assay in TNBC

Assay	Companion drug	Applicable population	Tumor cell interpretation criteria	Immune cell interpretation criteria	Positive threshold
22C3 (Dako)	Pembrolizumab	Locally recurrent unresectable or metastatic TNBC	CPS	CPS	CPS ≥ 10
SP142 (Ventana)	Atezolizumab	Advanced TNBC	Not evaluated	IC	IC ≥ 1%
JS311	Toripalimab	newly diagnosed metastatic or recurrent locally advanced TNBC	CPS	CPS	CPS ≥ 1
28–8 (Dako)	Nivolumab	Not applicable for breast cancer	None	None	None
SP263 (Ventana)	Durvalumab	Not applicable for breast cancer	None	None	None
**Scoring Method**					
IC (%) = $\frac{\text{Area of tumor infiltrated by PD-L1-stained immune cells}}{\text{Total tumor area}}$ × 100% (for SP142)					
CPS (%) = $\frac{\text{Number of PD-L1-stained cells (tumor cells, lymphocytes and macrophages)}}{\text{Total number of viable tumor cells}}$ × 100% (for 22C3)					

Abbreviations: *CPS* combined positive score, *IC* immune cell, *TNBC* triple-negative breast cancer

### TILs

TILs are a type of immune cells comprising mainly various subsets of T cells that infiltrate and accumulate within the TME and play a crucial role in the immune response against cancer by recognizing and targeting tumor-associated antigens. TNBC and HER2+ breast cancers exhibit elevated levels of TILs compared to luminal breast cancers, and high TILs have been reported to correlates with favourable prognostic outcomes [101]. Furthermore, a higher abundance of TILs indicates improved clinical outcomes in early-stage disease, regardless of the treatment regimen, especially in TNBC [103–106]. In the NeoPACT study, patients with high TILs achieved better pCR rates [73]. Of note, TILs could serve as a predictive biomarker for assessing response to immunotherapy for patients with mTNBC. As reported in the KEYNOTE-086 and KEYNOTE-119 trial, higher TILs resulted in a better clinical outcomes with pembrolizumab [15, 107]. Biomarker evaluation of the IMpassion130 study revealed improved outcomes with immunotherapy in PD-L1+ patients who were CD8-positive (CD8+) and stromal TIL-positive (TIL+) [108]. A meta-analysis suggested that high intra-tumoral, stromal, or invasive marginal CD8+ T cells, can predict clinical outcomes in patients with immunotherapy across different cancers [109]. FUTURE-C-Plus trial demonstrated an impressive ORR of 81.3% for patients with CD8+ immunomodulatory subtype mTNBC (CD8 IHC staining ≥10%) receiving the triple regimen of chemotherapy, PD1 antibody, and angiogenesis agent [110]. This positive result were validated in the subsequent FUTURE-SUPER trial, illustrating the potentially predictive ability of CD8 for immunotherapy [111]. A translational research assessed PD-L1 and TILs in Chinese TNBC multiomics dataset and two immunotherapy clinical trial cohorts. It was found that the TIL+/PD-L1- and TIL-/PD-L1+ groups were not typical “hot” tumors; both were associated with worse prognosis and lower immunotherapy efficacy than TIL+/ PD-L1+ tumors. Several genomic and transcriptomic alterations may lead to contradictory effects among TILs, PD-L1 expression, and prognosis in TNBC [112]. This study highlighted the potential clinical application of TILs and PD-L1 as combined biomarkers for TNBC.

### Microsatellite instability (MSI) / mismatch repair deficiency (dMMR)

The MMR system is responsible for recognizing and fixing errors that occur during DNA replication. Loss of functional mutations in MMR-related genes, such as MLH1, MSH2, MSH6, and PMS2, tend to damage the MMR system, leading to an accumulation of somatic mutations, especially in microsatellite sequences—short tandem repeats of DNA, a phenomenon known as MSI [113]. In the context of breast cancer, MSI/dMMR is associated with a higher mutation load and an increased proportion of neoantigens, and such neoantigens generated by the rapid accumulation of somatic mutations can act as targets for the immune system, making tumors with dMMR/MSI more responsive to ICI therapy [114, 115]. Based on data from five single-arm clinical trials (KEYNOTE-016/164/012/028/158), in May 2017, the FDA granted accelerated approval the use of pembrolizumab for adult and pediatric patients with unresectable or metastatic MSI-high (MSI-H) or dMMR solid tumors, who have progressed on prior therapy without satisfactory alternative treatment options, marking the first tumor-agnostic predictive biomarker approval [116]. It is worth noting that MSI-H is rarely present in breast cancers, approximately 1% of TNBC and less than 2% of all breast cancers are MSI-H, hence it remains uncertain whether it can be applied as a prognostic biomarker [117]. Moreover, MSI status can be detected directly by polymerase chain reaction or next-generation sequencing technology, or indirectly by IHC of the encoded protein from dMMR, but the results of different detection methods may have certain inaccuracy, and the detection standard needs to be unified [118]. Overall, the predictive worth of MSI on the benefit derived from immunotherapy in breast cancer merits further practice.

### TMB

TMB is defined as the count of somatic mutations in the tumor genomic coding region [119]. Typically, tumors with high TMB (TMB-H) tend to generate more neoantigens, which are more readily recognized by the immune system, thus inducing a more potent innate immune response. TMB-H has proven to be a predictive biomarker of positive outcomes in patients treated with ICI therapy across diverse tumor types, such as melanoma, NSCLC, and colorectal cancers [120–122]. In 2020, based on KEYNOTE-158, pembrolizumab was granted accelerated approval by the FDA for patients with unresectable or advanced-stage TMB-H [≥10 mutations/megabase (mut/Mb)] solid tumors, providing an alternative treatment option for TNBC patients with TMB-H who have progressed on prior multi-line therapy [123]. In addition, TMB represents tumor heterogeneity and immunogenicity, which varies across breast cancer subtypes, with TNBC and HER2+ tumors harboring relatively high mutation rates, while TMB-H is uncommon in breast cancer. An analysis of 3969 breast tumor samples estimated the overall rate of about 5% TMB-H tumors, with a slightly higher incidence at metastatic sites compared to primary sites [75]. TMB-H was related to improved outcomes in patients with breast cancer treated with immunotherapy, however, this benefit may be dependent on other tumor properties, such as PD-L1 status [125]. Confusingly, the predictive role of TMB for TNBC patients or their response to ICI therapy appears to be contradictory. A study of TNBC revealed significantly prolonged PFS (12.5 vs 3.7 months; *P* = 0.04) in patients with higher TMB (≥10 nonsynonymous mut/Mb) [126]. Conversely, an analysis of 10,000 breast cancer cases from the Cancer Genome Atlas failed to endorse this positive correlation. In the TNBC subgroup, no objective response was noted in TMB-H tumors, whereas the ORR in tumors with lower TMB was 20.5% [127]. TNBC patients with TMB-H in the GeparNuevo trial exhibited increased pCR rates with both neoadjuvant durvalumab plus chemotherapy and chemotherapy alone [128]. However, subsequent studies found that TNBC patients with low-TMB may benefit more from neoadjuvant durvalumab with longer DDFS, while adding durvalumab to chemotherapy did not improve outcomes in TNBC patients with TMB-H [129]. Another study found that mTNBC patients with low blood-based TMB (bTMB) (<6.7 mut/Mb) showed better response to the combination of a PD-L1 antibody TQB2450 and an angiogenesis inhibitor anlotinib (50% vs 7%; *P* = 0.015) and achieved greater PFS benefits (7.3 vs 4.1 months; *P* = 0.012) than those with high bTMB (≥6.7 mut/Mb) [130]. Additionally, the lack of standardized methods for determining TMB and additional data are required to determine a TMB cutpoint exclusively for breast cancer.

**Fig. 2:**
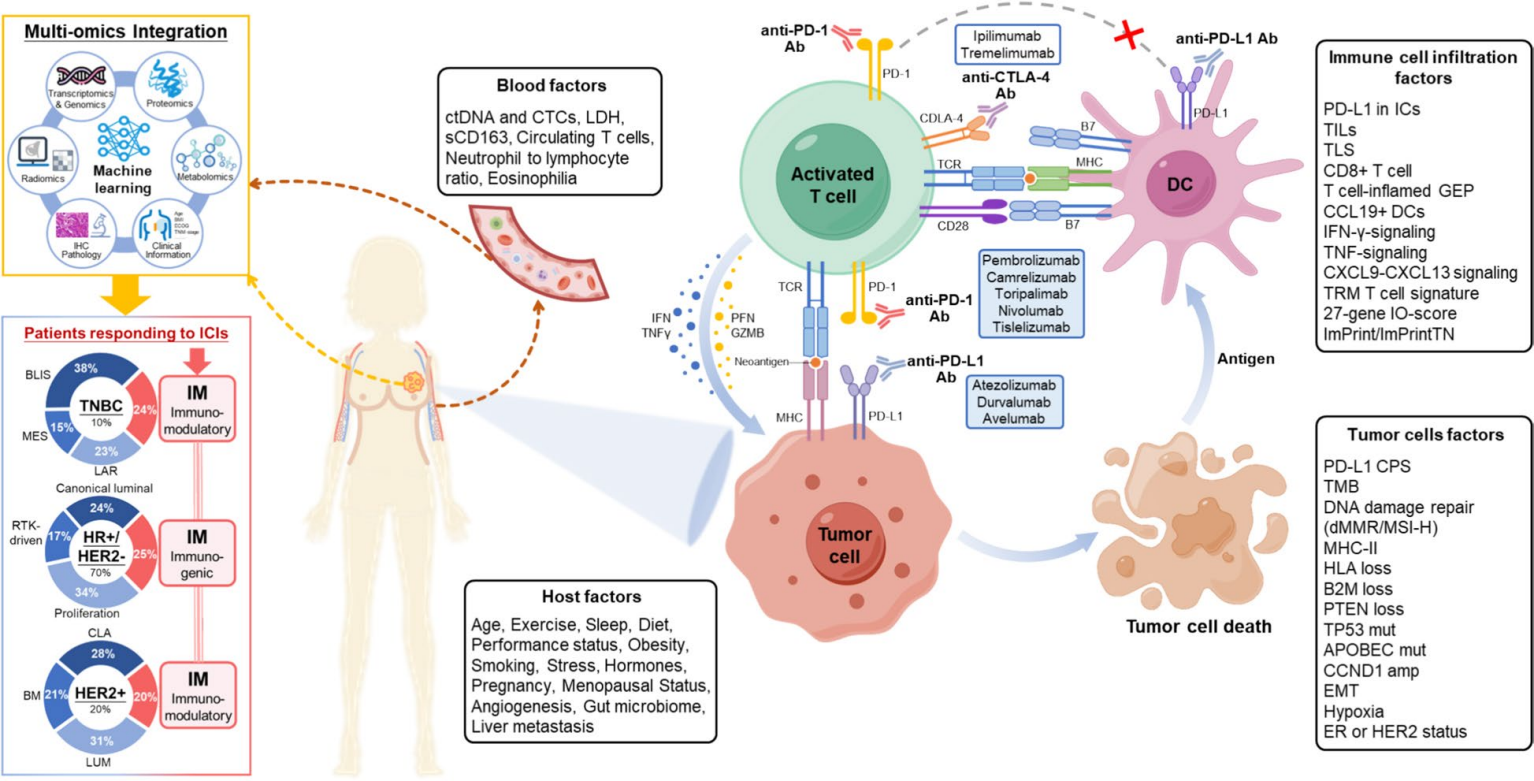
Factors related to immunotherapy response and multi-omics integration to explore immunotherapy benefit population in breast cancer. Shao et al. performed an integrated multi-omics profiling of the three traditional breast cancer subtypes TNBCs, HER2 + and HR + /HER2–, and reclassified them into four subtypes, of which immunomodulatory and immunogenic subtypes, featuring enriched immune cells and immune-activated microenvironment, potentially benefit from ICI therapy [131–133]. Intrinsic and extrinsic features of cancer cells, including host immunity, can influence the response to ICIs targeting the PD-1/PD-L1 axis. Comprehensive evaluation of such factors may be pivotal to select patients that are more likely to respond to ICI [134]. Abbreviations: *Ab* antibody, *ACTs* adoptive cell therapies, *Amp* amplification, *B2M* β2-microglobulin, *BLIS* basal-like immune-suppressed, *BM* basal/mesenchymal-like, *CPS* combined positive score, *CTC* circulating tumor cells, *ctDNA* circulating tumor DNA, *DCs* dendritic cells, *dMMR* mismatch repair deficiency, *CLA* classical, *ICs* immune cells, *ECM* extracellular matrix, *EMT* epithelial-mesenchymal transition, *ER* estrogen receptor, *GEP* gene expression profile, *GZMB* Granzyme B, *HER2* human epidermal growth factor receptor 2, *HR* hormone receptor, *HLA* human leukocyte antigen, *ICI* immune checkpoint inhibitor, *IHC* immunohistochemistry, *IFN* interferon, *IDO1* indoleamine2,3-dioxygenase1, *LAG-3* lymphocyte-activation gene 3, *LAR* luminal androgen receptor, *LDH* lactate dehydrogenase, *LUM* luminal-like, *MES* mesenchymal-like, *MHC* major histocompatibility complex, *Mut* mutation, *MSI-H* high microsatellite instability, *Mφ* macrophage, *PDO* patient-derived organoids, *PD-L1* programmed death-ligand 1, *PDX* patient-derived xenografts, *PFN* perforin, *sCD163* soluble variant of CD163, *SCNAs* somatic copy number alteration, *RTK* receptor tyrosine kinase, *TCR* T cell receptor, *TIL* tumor-infiltrating lymphocyte, *TLS* tertiary lymphoid structures, *TMB* tumor mutational burden, *TNBC* triple-negative breast cancer, *TNF* tumour necrosis factor, *TRM* tissue-resident memory

Despite progress, major challenges remain in establishing reliable biomarkers for breast cancer immunotherapy. Tumor heterogeneity, variability in immune microenvironments, and differences in biomarker testing methodologies pose obstacles to clinical translation. Future studies should focus on refining biomarker assays, validating findings across diverse patient populations, and integrating artificial intelligence (AI) to analyze complex multi-omics datasets. A multi-parameter approach combining several biomarkers may ultimately provide a robust predictive model for patient stratification in breast cancer immunotherapy. Recent advances in breast cancer immunophenotyping and biomarkers are summarized in Fig. 2.

## Novel combination strategies and future directions

**Fig. 3:**
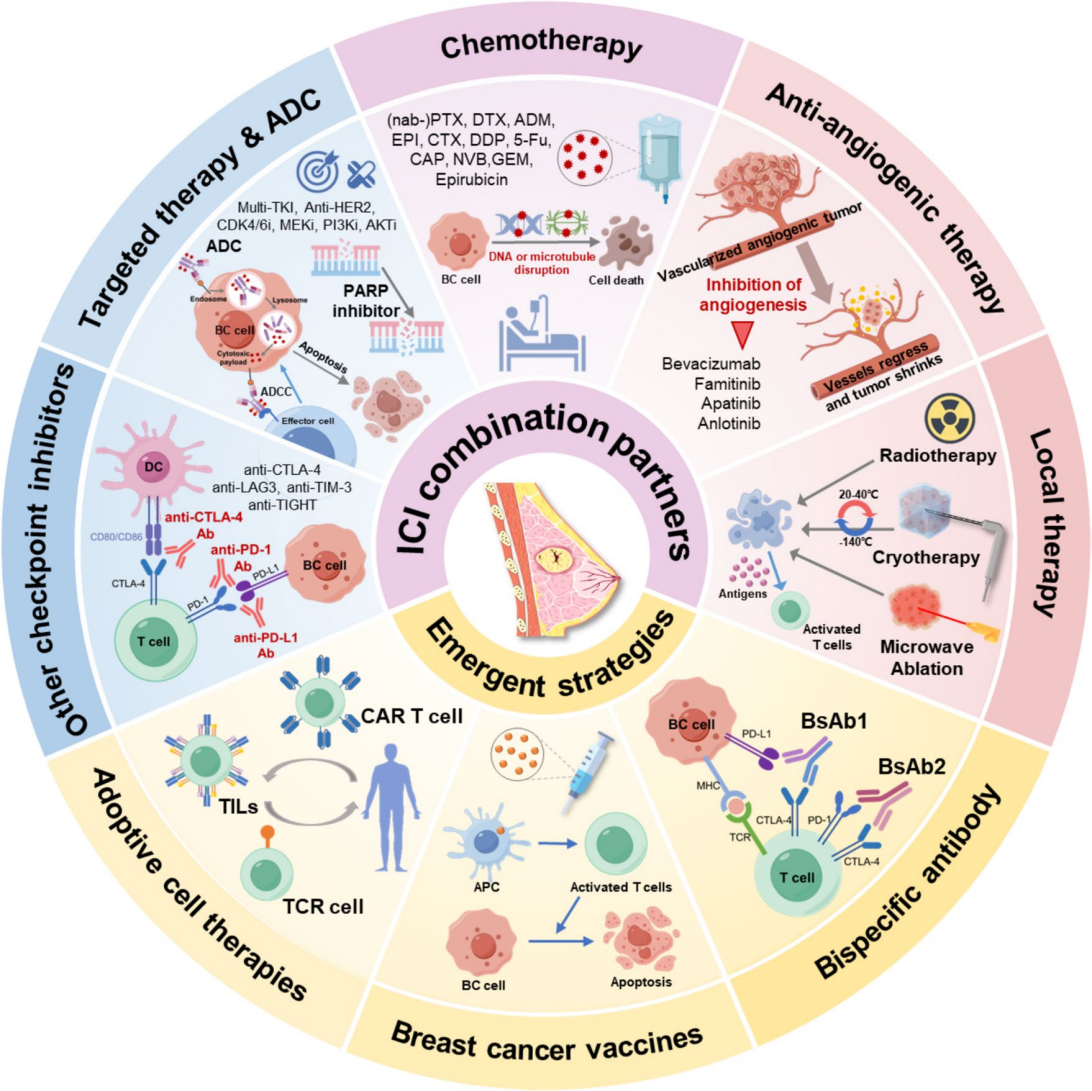
Combination partners for ICI and emerging immunotherapy strategies. Abbreviations: *Ab* antibody, *ADC* antibody–drug conjugate, *ADCC* antibody-dependent cell-mediated cytotoxicity, *ADM* doxorubicin, *AKTi* AKT inhibitor, *APC* antigen-presenting cell, *BC* breast cancer, *BsAb* bispecific antibody, *CAP* Capecitabine, *CAR T cell* chimeric antigen receptor T cell, *CDK4/6i* cyclin-dependent kinase 4/6 inhibitor, *CTLA-4* cytotoxic T-lymphocyte–associated protein 4, *CTX* cyclophosphamide, *DDP* cisplatin, *DTX* docetaxel, *EPI* epirubicin, *5-Fu* 5-Fluorouracil, *GEM* gemcitabine, *HER2* human epidermal growth factor receptor 2, *ICI* immune checkpoint inhibitors, *LAG-3* lymphocyte-activation gene 3, *MHC* major histocompatibility complex, *NVB* vinorelbine, *PARP* poly (ADP-ribose) polymerase, *MEKi* mitogen-activated extracellular signal-regulated kinase inhibitor, *TKI* tyrosine kinase inhibitors, *PD-L1* programmed death-ligand 1, *PD-1* programmed cell death protein, *PTX* paclitaxel, *TIM-3* T cell immunoglobulin and mucin domain-3 protein, *TIGIT* T cell immunoglobulin and ITIM domain, *TILs* tumor infltrating lymphocytes, *TCR* T cell receptor

Several ongoing trials are evaluating novel immunotherapy strategies, including combinations of ICIs with other treatments such as targeted agents, ADCs, angiogenesis inhibitors, other checkpoint inhibitors, radiotherapy and cryotherapy, as well as BsAbs, ACTs, and breast cancer vaccines (Fig. 3). Although these emerging strategies are not currently approved, they may develop into viable options for breast cancer patients shortly.

### ADCs

ADCs integrate the precision targeting capabilities of monoclonal antibodies with the potent cytotoxicity of chemotherapy agents, allowing the highly specific delivery of toxins to cancer cells while minimizing harm to surrounding healthy tissues. In addition to the previously mentioned landmark T-DXd and SG, Datopotamab deruxtecan (Dato-DXd), a TROP2-directed ADC, significantly improved PFS in HR+/HER2– MBC following the phase III TROPION-Breast01 trial [135]. Preclinical studies have suggested that the combination of ADCs with ICIs has the potential to promote the effectiveness of ICIs through direct activation of dendritic cells (DCs), upregulation expression of PD-L1 and the induction of neoantigen generation [136–138]. BEGONIA is an ongoing 2-part, phase II study evaluating durvalumab combined with other novel therapies as first-line treatment for mTNBC. A total of 62 patients were recruited in BEGONIA arm 7 and treated with Dato-DXd and durvalumab, which showed a favorable response with an ORR of 79%, a PFS of 13.8 months and a DoR of 15.5 months. Response to treatment was independent of PD-L1 expression level [139]. In BEGONIA arm 6, 56 patients HR-/HER2-low MBC received T-DXd plus durvalumab (34 ongoing) and 46 were included in the efficacy analysis, with a confirmed ORR of 56.5% [140]. The phase Ib/II MORPHEUS-pan BC (NCT03424005) study investigated atezolizumab plus SG or nab-paclitaxel in individuals with PD-L1+ mTNBC. Interim analyses reported higher ORR (76.7% vs 66.7%) and CBR (83.3% vs 66.7%) in the atezolizumab plus SG group than the control group, and PFS data are not yet mature but show a trend towards benefit [141]. Combined with the results of the BEGONIA trial, it is evident that the combination of ADC with PD-1/PD-L1 antibodies holds great promise.

In the neoadjuvant setting, 106 patients in the I-SPY 2.2 trial were randomly assigned to receive Dato-DXd plus durvalumab, of whom 53 achieved pCR with an overall pCR rate of 50%. This combination showed high pCR rates in immune(+) subtype (79%) and TNBC (62%) [142]. TROPION-Breast04 is evaluating Dato-DXd plus durvalumab as neoadjuvant treatment for TNBC and HR-low, HER2-low or negative EBC. TROPION-Breast05 is investigating Dato-DXd alone or combined with durvalumab in patients with PD-L1+ mTNBC. For TNBC patients with residual disease in the post neoadjuvant setting, two ongoing phase III studies, ASCENT-05/OptimICE-RD and TROPION-Breast03, will evaluate the addition of sacituzumab to pembrolizumab-based adjuvant therapy and Dato-DXd combined with durvalumab as adjuvant treatment, respectively [143, 144].

Other ongoing clinical trials testing the combination of ADCs and ICIs include: T-DXd plus pembrolizumab (NCT04042701); SG plus pembrolizumab (NCT04468061); ladiratuzumab vedotin plus pembrolizumab [145].

### Poly (ADP-ribose) polymerase inhibitors

Poly (ADP-ribose) polymerase (PARP) inhibitors have demonstrated remarkable therapeutic benefits in patients harboring germline BRCA1/2 mutations. Preclinical data indicated that PARP inhibitors may stimulate the host antitumor immune responses by generating neoantigens by DNA damage, upregulating interferon synthesis via the STING pathway, and augmenting PD-L1 expression [146, 147]. The phase I/II MEDIOLA trial suggested that olaparib plus durvalumab achieved an ORR of 58.5% and a PFS of 8.2 months in germline BRCA-mutated HER2- MBC [148]. In the phase I/II TOPACIO/KEYNOTE-162 trial, pembrolizumab plus niraparib yielded an ORR of 47% in BRCA-mutated mTNBC with good tolerability [149].

For mTNBC maintenance therapy, the phase II/III KEYLYNK-009 study found pembrolizumab plus olaparib as maintenance therapy did not lead to a notable enhancement in PFS and OS compared with pembrolizumab plus chemotherapy, including those with a PD-L1 CPS ≥10 and tumor BRCA mutations (tBRCAm). However, pembrolizumab plus olaparib numerically prolonged PFS in tBRCAm patients. In addition, the olaparib group had a lower incidence of treatment‐related adverse events (TRAEs) [150]. The phase II DORA study evaluated olaparib alone or combined with durvalumab as a de-chemotherapy maintenance therapy for platinum-sensitive patients with mTNBC. Median PFS was 4.0 months with olaparib and 6.1 months with the combination, both significantly surpassing historical control. CBR were 44% and 36% in the monotherapy and combination groups, respectively. Durable clinical benefit was observed regardless of germline BRCA mutation or PD-L1 status [151].

### Angiogenesis inhibitors

Abnormal tumor-associated neovascular systems have been proven to induce various immunosuppressive features, and antiangiogenic therapy can improve antitumor immunity [152, 153]. The single-arm phase II NEWBEAT study evaluated nivolumab + paclitaxel + bevacizumab [an anti-vascular endothelial growth factor (VEGF) antibody] as first-line treatment in HER2- MBC patients. The ORR was 70% for all patients, 74% for HR+ patients and 59% for TNBC patients. PFS and OS were 14.0 and 32.5 months, respectively [154]. In the single-arm phase II FUTURE-C-Plus trial, patients with immunomodulatory subtype mTNBC received a triple combination of famitinib (an antigiogenesis inhibitor) + camrelizumab + nab-paclitaxel in the first-line setting, yielding a respectable and durable response, with an ORR of 81.3%; PFS and OS reached 13.6 months and 29.4 months, respectively [155, 156]. Subsequently, the randomized phase II FUTURE-SUPER trial confirmed an absolute benefit in PFS of 8.6 months in the immunomodulatory cohort receiving this triple regimen versus nab-paclitaxel alone (15.1 vs 6.5 months; hazard ratio, 0.46) [111]. The phase III trial assessing the efficacy of this triple regimen for immunomodulatory mTNBC (NCT05760378) is recruiting. For patients with pretreated mTNBC, a phase II trial (NCT04303741) displayed a promising efficacy of camrelizumab + apatinib + eribulin, with an ORR of 37.0%, a DCR of 87.0% and a PFS of 8.1 months [157]. Moreover, two clinical studies exploring chemotherapy-free regimens of ICI plus antigiogenesis inhibitor exhibited favorable antitumor activitiy and a manageable safety profile in pretreated mTNBC. In a phase II trial (NCT03394287), patients who received camrelizumab with apatinib continuous dosing had an ORR of 43.3%, a DCR of 63.3%, and a PFS of 3.7 months [158]. In a phase Ib trial (NCT03855358), patients who received benmelstobart (TQB2450, an anti-PD-L1 antibody) plus anlotinib (an anti-angiogenic inhibitor), experienced a ORR of 26.5% and a PFS of 5.6 months [159].

### PI3K or AKT inhibitors

The phase II MARIO-3 trial indicated that eganelisib (a PI3Kγ Inhibitor) combined with atezolizumab and nab-paclitaxel as first-line therapy for TNBC exhibited promising efficacy (ORR, 55.3% and DCR, 84.2%) regardless of PD-L1 status, with manageable toxicity. Two randomized phase II trials, LOTUS [160] and PAKT [161], evaluated ipatasertib and capivasertib, respectively, and both demonstrated that AKT inhibitors combined with paclitaxel as first-line treatment improved PFS in mTNBC. Preclinical evidence suggested that AKT inhibitors may boost the efficacy of ICIs by retaining a stem-like phenotype in memory T cells, preventing exhaustion and allowing patients to achieve long-term responses [162, 163]. A pooled analysis that included the phase Ib CO40151 study, the C cohort of IPATunity130, and the phase III IPATunity170 trial examined a triplet regimen combining atezolizumab, ipatasertib, and (nab-)paclitaxel as first-line therapy for mTNBC. Among 317 patients treated with the triplet, PFS was 5.4 to 7.4 months, ORR was 44–63%, DoR was 5.6 to 11.1 months, and OS was 15.7 to 28.3 months. Biomarker analysis revealed that patients with high baseline predicted pAKT-S473 levels had low immune infiltrates with a trend toward longer PFS in these patients, suggesting that ipatasertib may have improved immune infiltration and synergized with atezolizumab for patients whose tumors were immune excluded and had low PD-L1 levels [164].

### MEK inhibitors

Previous studies have indicated that the use of mitogen-activated extracellular signal-regulated kinase (MEK) inhibitors may help to overcome taxane resistance [165, 166]. In the phase II COLET study, Cohorts II and III explored the combination of atezolizumab, cobimetinib (a MEK1/2 inhibitor) and (nab-)paclitaxel as first-line treatment for patients with mTNBC. 32 and 31 patients were randomized 1:1 to receive atezolizumab, cobimetinib, and paclitaxel (Cohort II) or atezolizumab, cobimetinib, and nab-paclitaxel (Cohort III). Cohort II/III had an ORR, DOR, and PFS of 34.4%/29.0%, 5.8/11.0 months and 3.8/7.0 months, respectively. Patients with PDL1+ (IC ≥1%) tumors showed a trend towards improved ORR and PFS compared to those with PDL1- (IC <1%) tumors (ORR, 39% vs 20%; PFS, 7.0 vs 3.7 months), but the number of patients was small and should be interpreted with caution [167].

### Other immune targets and dual immunotherapy

With the great success of PD-(L)1 inhibitor in breast cancer, a growing number of trials reported preliminary efficacy against other immune checkpoints. Tremelimumab is a cytotoxic T-lymphocyte-associated protein 4 (CTLA-4)-targeted humanized IgG2 monoclonal antibody, which inhibits tumor growth by preventing the interaction between CTLA-4 and B7s and thereby allowing T-cell activation [168]. A phase I study evaluating tremelimumab plus exemestane in 26 patients with HR+ MBC revealed a tolerable safety profile, and a best overall response of SD ≥12 weeks in 11 patients [169]. In a pilot study, the combination of tremelimumab and durvalumab exhibited poor outcomes in overall MBC patients; however, TNBC patients achieved better clinical benefits than ER+ patients (ORR, 43% vs 17%). Unexpectedly, TNBC patients who received durvalumab monotherapy had a superior response [170]. Ipilimumab is another anti-CTLA-4 antibody, with proven antitumor activity against various cancers [171]. A single-arm phase II trial examined the addition of ipilimumab and nivolumab to neoadjuvant paclitaxel in stage III eTNBC. The pCR rate was 24.2%, 37.5% in PD-L1+ and 23% in PD-L1- patients, and the ORR in the breast was 57.6%. However, the incidence of low-grade pneumonitis is relatively high [172]. In the phase II adaptive BELLINI trial, 30 patients with eTNBC recieved 4 weeks of neoadjuvant nivolumab or nivolumab plus ipilimumab before regular therapy, experienced immune activation in 53% and 60% of patients with eTNBC, respectively. Higher TILs and higher baseline proportion of CD8+ T cells were found to be associated with response. Patients with high TILs (≥50%) received another 6 weeks of neoadjuvant nivolumab plus ipilimumab followed by surgery. Overall, 53% of patients achieved a major pCR (<10% viable tumor), and 33% achieved a pCR. However, 17% of patients developed grade ≥3 AEs and 57% developed immune-mediated endocrine disease. This study revealed that short-term neoadjuvant nivolumab with or without ipilimumab, could induce immune activation and lead to pCR and ctDNA clearance in the most patients, and this dual immunotherapy may be a promising chemo-free regimen for eTNBC patients [173]. The phase II Nimbus trial assessed the combination of ipilimumab and nivolumab in 30 patients with TMB-H HER2– MBC. This study achieved the primary endpoint and demonstrated a ORR of 13.3%. PFS and OS were 1.4 and 8.8 months, respectively. Exploratory analysis did not show a difference in ORR according to HR status and PD-L1 status, but patients with TMB ≥14 mut/Mb had a higher ORR of 60% versus 4% in the patients with TMB between ≥9 and <14 mut/Mb (*P* = 0.01) [174]. For TNBC patients with residual disease following NACT, the phase II BreastImmune-03 trial reported at SABCS 2024 indicated that 6-month postoperative nivolumab plus ipilimumab did not show advantages compared to 8 cycles of capecitabine. Regrettably, this trial was prematurely terminated due to TRAEs [175].

Some agents targeting additional immune checkpoints are gradually being discovered to block T cell suppression, including the lymphocyte-activation gene 3 (LAG-3), T cell immunoglobulin and mucin domain containing-3 (TIM-3), T cell immunoglobulin and ITIM domain (TIGIT), and the T cell agonist such as OX-40 (CD134) and 4-1BB [176, 177]. LAG-3 is a novel inhibitory receptor, highly expressed in regulatory and disabled T cells. Unlike PD-1/PD-L1 and CTLA-1 blockers, LAG3 blockers can not only inhibit regulatory T cells (Tregs) but also enhance effector T-cell activity [178]. IMP321 is a soluble LAG-3Ig that binds to MHC class II with high avidity and mediates antigen-presenting cell activation followed by CD8 T cell activation [179, 180]. A phase I/II trial (NCT00349934) reported that IMP321 plus paclitaxel as first-line treatment achieved an ORR of 50%, superior to historical controls group of 25% [181]. The phase IIb AIPAC study (NCT02614833) indicated that IMP321 plus weekly paclitaxel did not prolong median PFS in patients with HR+/HER2- MBC. However, IMP321 is well tolerated and induced a sustained increase in CD8 T cells in blood and clinical benefits in some subgroups [182]. AIPAC-003 is an ongoing phase III trial testing IMP321 plus paclitaxel versus placebo plus paclitaxel in patients with HR+/HER2-negative or low and endocrine therapy-resistant MBC.

At present, clinical trials are actively investigating anti-LAG-3 combined with anti-PD-1 (NCT03250832), anti-PD-1 with anti-TIGIT (NCT03628677), and anti-PD-L1 with anti-B7-H4 (NCT03514121). Notably, patients treated with dual ICI may experience increased toxicity due to the simultaneous blockade of various immunosuppressive mechanisms. The potential for significant immune-related toxicity must be considered before applying treatment regimens with multiple immunological agents.

### Bispecific antibodies

Research on bispecific antibodies (BsAbs) or bispecific T-cell engager (BiTE) antibodies has developed tremendously over the past decades. BsAbs are engineered recombinant proteins designed to target two specific antigens. BiTE is a special BsAb that binds tumor-cell-specific antigen and cytotoxic T cells by binding and activating CD3 via a special engager to mediate cancer cell lysis [183].

PRS-343, the first HER2/4-1BB bispecific antibody to enter human trials, reported a 40% ORR and a 70% DCR (10% CR, 30% PR) in a phase I study (NCT03330561) of 70 patients with HER2+ solid tumor (16 breast cancer), with no serious AEs [184]. Bintrafusp alfa (M7824) is a bifunctional fusion protein composed of the extracellular domain of the TGF-β receptor II (a TGF-β “trap”) fused to a human IgG1 monoclonal antibody blocking PD-L1 [185]. In an ongoing phase I trial (NCT02517398), 33 patients with heavily pretreated mTNBC were treated with bintrafusp alfa. Interim results revealed 1 CR, 2 PR, and 5 cases of disease control, with a median OS of 7.8 months [186]. A phase II trial of bintrafusp alfa (NCT04489940) is ongoing.

Another BsAbs strategy is engineering the molecules to simultaneously target two distinct key immune checkpoint molecules (eg. PD-L1 and CTLA-4), thereby reducing the development of resistance to immunotherapies. In the phase II study (NCT03872791), KN046 (a PD-L1/CTLA-4 bispecific antibody) plus nab-paclitaxel as first-line treatment for mTNBC patients achieved a 44.0% ORR, a 7.33-month PFS, and a 30.92-month OS. PD-L1+ patients benefited more from this combination therapy compared to PD-L1- patients (PFS, 8.61 vs 4.73 months; 2-year OS rate, 62.5% vs 57.1%), and it was generally well tolerated [187]. An ongoing phase II trial (NCT04521179) assessed the combination of KN046 and KN026 (a BsAb that binds to two different HER2 epitopes shared by trastuzumab and pertuzumab) in heavily pretreated patients with HER2+ MBC. 36 patients were enrolled and 22 patients were evaluable for overall response. The ORR was 50.0% (1 CR), and DCR was 81.8%, with PFS of 5.6 months. This chemotherapy-free regimen provided favorable clinical efficacy with manageable side effects [188]. Other PD-L1/CTLA-4 BsAbs such as XmAb20717 (NCT03517488) and SI-B003 (NCT04606472) are under investigation in solid tumors. A phase Ib/II study of PM8002 (a BsAb targeting PD-L1 and VEGF-A) plus nab-paclitaxel presented acceptable safety and encouraging antitumor activity in 42 previously untreated mTNBC patients. The ORR was 78.6% (1 CR and 32 PR), DCR was 95.2%, DoR was 7.2 months and PFS was 9.2 months. Subgroup analyses showed a trend toward improved efficacy with increasing PD-L1 expression [189]. MGD013 (NCT04178460) and tebotelimab (NCT03219268), both BsAbs targeting PD-L1 and LAG-3 are underway in phase I trials. Nowadays, engineered BsAbs are no longer confined to targeting tumor cells or T cells. Other inflammatory cells such as natural killer (NK) cells or macrophages can be targeted by BsAbs [190]. BsAbs can also target multiple activation signals simultaneously, such as DF1001 targeting NK and T cell activation signals against HER2+ solid tumors (NCT04143711).

### Adenosine receptor inhibitors

Adenosine, an immunosuppressive metabolite abundant in the TME, is significantly produced due to hypoxia, rapid cell turnover, and the expression of CD39 and CD73 enzymes [191]. Metabolic reprogramming have been implicated in the emergence of treatment resistance in breast cancer [192, 193]. The activation of adenosine receptors, such as A2aR and A2bR, can suppress T cell proliferation, cytokine production, and cytotoxicity [194]. In a phase Ib trial, CPI-444, an adenosine A2aR inhibitor, demonstrated clinical activity both as monotherapy or combined with atezolizumab in patients with advanced solid tumors, including TNBC [195]. The phase II SYNERGY trial explored durvalumab, paclitaxel, and carboplatin with or without the anti-CD73 antibody oleclumab a first-line therapy in 127 patients with mTNBC. The results revealed that the addition of oleclumab to durvalumab with carboplatin/paclitaxel did not improve CBR at week 24 and PFS among mTNBC patients [196].

### Local therapy

Local therapies such as radiation therapy and cryotherapy are immunogenic and have the potential to optimize the response to immunotherapy. Strategies for combining these therapies with ICI are actively under investigation.

### Radiotherapy

Several studies have investigated the efficacy of radiotherapy combined with ICI in breast cancer. A phase II trial examined pembrolizumab plus radiotherapy in 17 patients with heavily pretreated mTNBC who were unselected for PD-L1 status. The result revealed an ORR of 17.6% across the entire cohort, with responders experiencing a complete reduction in tumor volume outside the irradiated portal [197]. Another phase II study examined pembrolizumab plus palliative radiotherapy in patients with heavily pretreated HR+/HER2- MBC, resulting in no objective responses and stopping enrollment after the first cohort [198]. A single-institution study explored the safety and efficacy of brain radiotherapy and concurrent tremelimumab-mediated CTLA-4 blockade with or without trastuzumab for patients with breast cancer brain metastases. The regimen was tolerated and showed not sufficiently active in HER2- disease. However, SD and a PR lasting >6 months were observed in patients with trastuzumab-refractory HER2+ MBC receiving tremelimumab and brain radiotherapy plus trastuzumab [199]. The phase II Neo-CheckRay trial evaluated stereotactic body radiotherapy (SBRT) +/– durvalumab +/– oleclumab together with NACT for 147 patients with ER+/HER2- EBC. The addition of durvalumab +/– oleclumab numerically increases pCR (35.6% vs 17.8%) and RCB 0/1 rates (51.1% vs 37.8%) compared to NACT + SBRT [200]. A pilot study (NCT05132790) suggested that neoadjuvant SBRT plus adebrelimab and chemotherapy resulted in a considerable pCR rate of 90% in eTNBC patients [201]. Further research is imperative to clarify the optimal dose, fractionation, and timing of radiotherapy delivery concerning ICI. Accurate patient selection is essential to ensure benefit with the combination of immunotherapy and radiotherapy.

### Cryotherapy

A pilot study demonstrated that preoperative cryoablation and/or ipilimumab (10 mg/kg) were safe and well-tolerated for 19 patients with operable breast cancer, without delaying planned mastectomy. The combination therapy induced systemic and intratumoral immune activation, including enhanced Th1-type cytokines, proliferating T cells, and an increased Teff/Treg ratio, suggesting potential synergistic antitumor immunity [202]. The same research group then conducted a small pilot study investigating dual checkpoint blockade with cryotherapy, comprising single-dose ipilimumab, nivolumab, and cryoablation in five patients with EBC. All five patients underwent standard-of-care surgery without delay, meeting the primary endpoint of safety [203]. The results from these pilot studies prompted the expansion of this strategy into a larger phase II study examining ipilimumab, nivolumab, and cryoablation in 80 TNBC patients (NCT03546686).

### Microwave ablation

Microwave ablation (MWA), as a minimally invasive local therapy, has been found in preclinical studies to have favorable efficacy when combined with ICI for the treatment of breast cancer [204–206]. A window-of-opportunity trial (NCT04805736) confirmed that preoperative camrelizumab plus MWA was feasible and safe in early-stage breast cancer. Sixty patients were randomized 1:1:1 to receive camrelizumab alone, MWA alone, or camrelizumab plus MWA. In the MWA group with or without camrelizumab, 34 of 37 evaluable samples achieved complete ablation (91.9%). Notably, this combination strategy enhanced peripheral CD8+ T cells response that depended on monocytes with activated MHC class I pathways. The long-term clinical benefit of this combination therapy merits further examination [207].

### Breast cancer vaccines

Anticancer vaccines are engineered to stimulate the immune system by activating antigen-specific T cells to recognize and eliminate cancer cells [208]. Extensive research has been conducted on peptide-based vaccines for breast cancer, and several of them, such as the E75, GP2, and AE37 vaccines against HER2, have exhibited potential clinical efficacy [209, 210]. In a phase I/II trial (NCT00841399/NCT00584789), E75 vaccine was well tolerated and reduced recurrence rates (5-year DFS: 89.7% vs 80.2%; *P* = 0.08) in breast cancer patients whose tumors expressed any level of HER2 (IHC 1–3+). However, a subsequent interim analysis of the phase III trial (NCT01479244) reported that E75 vaccine failed to significantly improve DFS compared to placebo, contributing to the early termination of the study [211, 212]. Another phase IIb trial (NCT01570036) suggested that E75 vaccine plus trastuzumab did not improve DFS in patients with high-risk HER2-low breast cancer compared to trastuzumab alone [213]. However, subgroup analyses revealed potential benefits in patients with TNBC or those who are HLA-A24 positive [214]. A meta-analysis including 24 clinical studies indicated that E75 vaccination significantly reduced disease recurrence rate and improved DFS, without a significant effect on OS [215]. Additionally, a phase I/II study found that a DC vaccine pulsed with two HER2 peptides given with trastuzumab and vinorelbine can induce immune responses in patients with HER2-overexpressing [216]. A phase IIa study is exploring a DC vaccine targeting HER2 and HER3 combined with pembrolizumab in patients with brain metastasis from TNBC or HER2+ breast cancer [217].

TNBC expresses a range of non-HER2 tumor-associated antigens (TAAs), with cancer-testis antigens (CTAs) emerging as frequent targets for cancer vaccination strategies [218]. Various prominent CTAs like New York esophageal squamous cell carcinoma 1 (NYESO-1), Wilms’ tumor protein (WT1), folate receptor alpha (FRα), melanoma antigen gene protein-12 (MAGE-12), brachyury protein, and p53 have been considered for the development of TNBC vaccines [219–221]. Nonetheless, CTA-targeted vaccines have not met the anticipated success requiring further clinical research.

PANVAC is a recombinant pox viral vaccine that targets the TAAs, Mucin 1 (MUC1) and CEA, and also contains three T-cell co-stimulatory molecules (B7.1, LFA-3, and ICAM-1). A phase II trial (NCT00179309) demonstrated that PANVAC plus docetaxel significantly improved PFS compared to docetaxel alone in patients with MBC of all subtypes (7.9 vs 3.9 months; hazard ratio, 0.65; 95% CI, 0.34-1.14; *P* = 0.09) [222].

Other vaccines against tumor-associated carbohydrate antigens, such as P10s-PADRE mimotope vaccine, are on testing in patients with stage I–III TNBC [223]. Moreover, a vaccine targeting the Globo H glycosphingolipid antigen has undergone clinical investigation in patients with distinct breast cancer subtypes. In a phase II trial (NCT01516307), the treatment involving a synthetic Globo H vaccine conjugated with adagloxad simolenin, along with the adjuvant OBI-821, was well-tolerated and displayed the induction of anti-Globo H humoral immune responses in patients with Globo H-positive MBC. The combination of adagloxad simolenin and OBI-821 has advanced to a phase III clinical trial (NCT03562637) in patients with Globo H-positive TNBC [224].

However, clinical trials evaluating breast cancer vaccines have offered limited evidence of clinical benefits despite the successful induction of immune responses. More research is warranted to explore the optimal immunization dose and schedule, delivery routes, and choices of immunologic boosters.

### ACTs

ACTs involve the isolation of T cells from peripheral blood or tumors, which are then modified, activated, and expanded ex vivo before being reintroduced into the patient [225]. ACTs are broadly categorized into three main types: TIL-based therapies, T cell receptor (TCR) gene therapy, and chimeric antigen receptor T cell (CAR-T cell) therapy [226]. These therapies work by harnessing the infused cells to recognize tumor-associated antigens and trigger a cytotoxic immune response. Initially, this technology was applied to advanced refractory hematological malignancies [227]. Currently, CAR-T cells are being extensively investigated among solid tumors, although their use in breast cancer is still primarily confined to phase I clinical trials [228]. In a phase II pilot trial (NCT01174121), infusion of autologous activated lymphocytes against specific tumor antigens was confirmed to induce a durable response in a patient with refractory MBC treated with mutation-specific TILs in combination with pembrolizumab [229]. In another study testing the feasibility of mRNA c-MET-CAR-T cells, only one of six patients with ER+/HER2- MBC had a best response of SD [230]. The development of ACTs in solid tumors faces a few challenges, including the heterogeneity of the antigenic milieu, harsh conditions within TME, and insufficient T cell infiltration into the tumor nests. Another obstacle lies in the toxicities related to lymphodepletion and immune-mediated adverse effects, particularly neurotoxicity and cytokine release syndrome, both posing significant and potentially fatal risks [231]. To counter these difficulties, several strategies are under development, and promising CAR-T cell targets such as HER2, MUC1, or mesothelin have been determined for breast cancer management [232]. The discovery of neoantigens and the availability of other immune cell types, such as NK cells or DCs offer new avenues for the advancement of ACTs.

## AI-enabled breast cancer immunotherapy

**Fig. 4:**
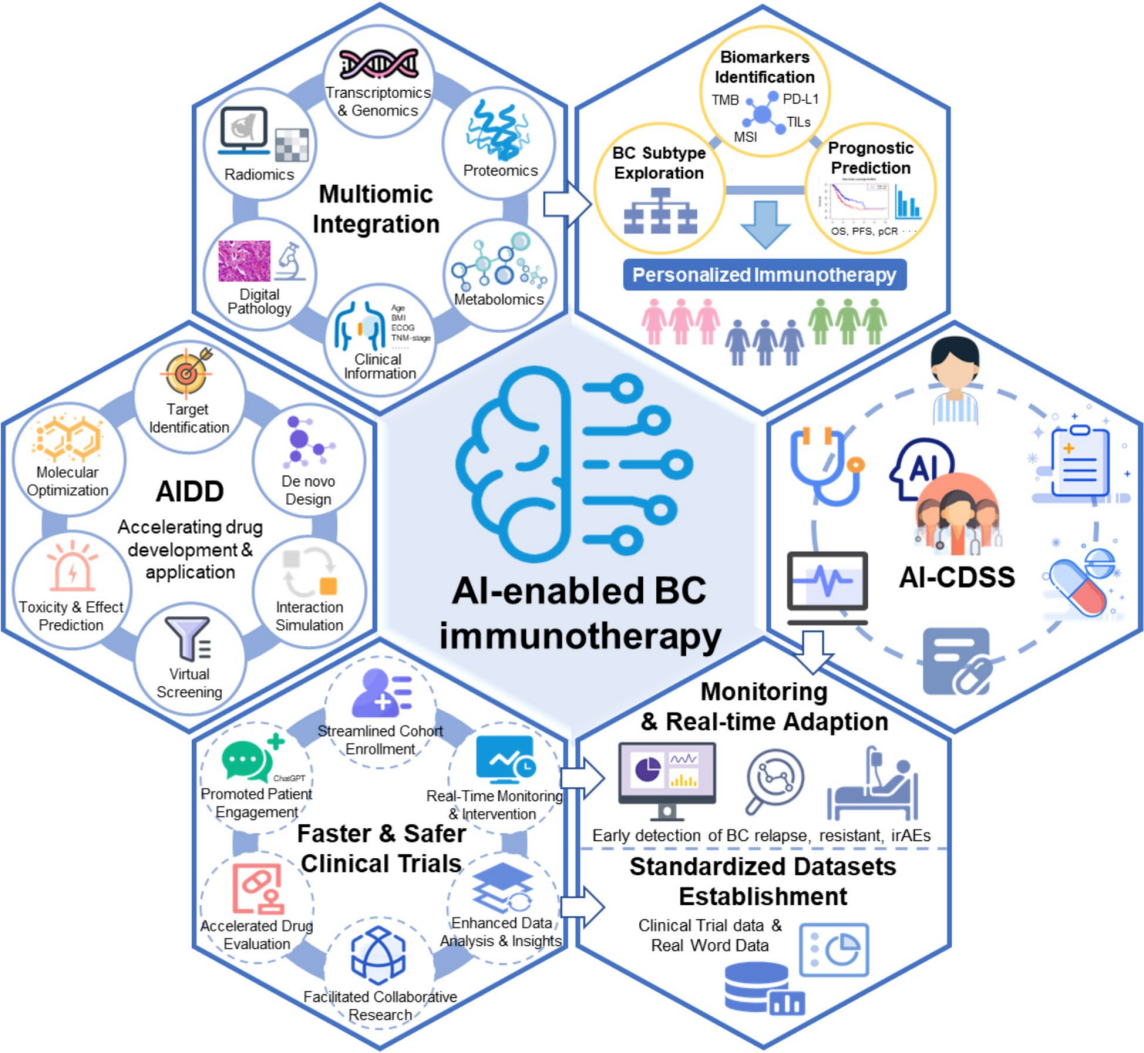
The application and prospects for AI in breast cancer immunotherapy. Abbreviations: *AI* artificial intelligence, *AI-CDSS* artificial intelligence-powered clinical decision support systems, *AIDD* artificial intelligence-driven drug design, *BC* breast cancer, *irAEs* immune-related adverse events, *MSI* microsatellite instability, *PD-L1* programmed death-ligand 1, *TILs* tumor infiltrating lymphocytes, *TMB* tumor mutational burden

In recent years, artificial intelligence (AI) including machine learning (ML) and deep learning, has emerged as a transformative force in the field of medical research and practice [233–240]. Currently in breast cancer immunotherapy, AI is mainly applied to assess the TME of breast cancer, identify relevant biomarkers and predict patient response to immunotherapy. Figure 4 depicts the application and prospects for AI in breast cancer immunotherapy.

PD-L1 is an important biomarker for identifying patients suitable for ICIs, but it lacks a clear gold standard and is subjective and semi-quantitative. There is a low rate of consistency and repeatability in the assessment of PD-L1 expression by different pathologists [241–243]. AI-based digital pathology reportedly have the potential to help pathologists improve diagnostic accuracy, efficiency and concordance. Two multi-institutional ring studies proposed an AI-assisted model based on deep-learning method that enabled pathologists at different levels to achieve good consistency and repeatability in interpreting PD-L1 (Dako 22C3) CPS and PD-L1 (SP-142) IC in breast tumor samples, respectively [244, 245].

Computational imaging methods derived from AI called radiomics automatically quantify the radiological features of tumors, providing more detailed information than the naked eye, displaying macroscopic, molecular, and cellular features. Radiomics-based prediction of ICI response has been successful in different types of tumors [246–249]. Zhao et al. developed the first radiomics model for immunotherapy response prediction in patients with MBC based on contrast-enhanced CT of 240 patients 1 month prior to ICIs-based therapies [250]. This non-invasive prediction model performed effectively both in the training and validation cohort. Moreover, the radiomics model could divide patients treated with ICIs therapies into high-risk and low-risk group with significantly different PFS. Subgroup analyses showed that the accuracy of this radiomics model was not influenced by PD-L1 status, TMB or molecular subtype, which could be widely applied to assist breast oncologists in making decisions of ICIs-based therapies for MBC patients.

The accumulation of patient demographics, medical history, imaging, pathology images, laboratory data, sequencing data and other comprehensive information laid a solid foundation of big data to analyse multi-omics data using AI for modelling and prediction in order to identify populations who will benefit from ICIs. Based on a multiomics dataset of 386 patients with TNBC, Xiao et al. performed an extensive immunogenomic analysis and used k-means clustering algorithm to classify the TME of TNBC into three clusters: immune-desert cluster, with low ICs infiltration; innate immune-inactivated cluster, with resting innate immune cells and nonimmune stromal cells infiltration, these two were regarded as “cold” tumors; and immune-inflamed cluster, with abundant adaptive and innate immune cells infiltration, which might be responsive to ICIs [251]. To provide a broader molecular profile of TNBC, this Chinese research team analyzed clinical, genomic, and transcriptomic data of 465 patients with TNBC using used k-means clustering algorithm and categorized TNBCs into four transcriptome-based subtypes: luminal androgen receptor (LAR), IM, basal-like immune-suppressed (BLIS), and mesenchymal-like (MES). The hallmarks of the IM subtype were elevated immune cell signaling and TILs, implying that patients with the IM subtype might benefit from ICIs [131]. On this basis, the team developed an IHC-based classification method that simplified and improved the clinical utility of the subtype system. The results for PD-1 blockade plus chemotherapy in the IM subtype showed the highest ORR, with a PFS of 4.6 months and an OS of 16.1 months [252]. This was also validated in the subsequent FUTURE-C-Plus and FUTURE-SUPER trials [110, 111]. Employing a similar strategy, this team constructed a large multiomics cohort of 579 Chinese patients with HR+/HER2– breast cancer, integrating analyses of their somatic copy number alterations, RNA sequencing and metabolome data. Four molecular subtypes were identified: canonical luminal, immunogenic, proliferative and receptor tyrosine kinase (RTK)-driven. The immunogenic subtype had enriched ICs and could benefit from ICI therapy. This team also developed convolutional neural network models through deep learning that can infer subtypes from pathology whole-slide images [132]. Recently, Jiang et al. further established a comprehensive multiomics cohort comprising 773 Chinese patients with breast cancer and systematically examined their genomic, transcriptomic, proteomic, metabolomic, radiomic and digital pathology features. Based on the transcriptomic data, three TME of breast cancer were categorized as “cold”, “moderate” and “hot”. The “hot” TME contained a large number of adaptive and activated intrinsic ICs, exhibited a strong anti-tumor response and the highest predictive score for immunotherapy efficacy. To accurately predict the risk of recurrence in patients with breast cancer, a multimodal model TMPIC was built using ML integrating transcription, metabolism, pathology, IHC, and clinical stage with a consistency index of 0.78, which was validated to be more precise than unimodality for prognostic stratification [253]. Researchers at the National Cancer Institute and Memorial Sloan Kettering Cancer Center have jointly developed an AI-driven ICI prediction model called LORIS using a six-feature [TMB, systemic therapy history, blood albumin, blood neutrophil-lymphocyte ratio, age and cancer type] logistic regression model. The investigators analyzed a large dataset of 2,881 ICI-treated (PD-1/PD-L1 inhibitor or CTLA-4 inhibitor or a combination of both) and 841 non-ICI-treated patients across 18 solid tumor types, including breast cancer. LORIS predicted not only patient ICI objective response but also short- and long-term survival benefit following immunotherapy better than established methods (RF16 [254]). More importantly, LORIS successfully identified patients with low TMB or PD-L1 expression who can still benefit from immunotherapy [255]. In another study, researchers developed a ML model called SCORPIO which utilized routine blood tests and basic clinical characteristics of 9745 ICI-treated cancer patients (21 cancer types, including breast cancer) from three real-world cohorts and 10 phase III clinical trial cohorts to predict outcomes after ICI administration. SCORPIO outperformed PD-L1 IHC and TMB in forecasting clinical benefit and OS, and was superior in predicting OS in real-world cohorts compared to phase III clinical trial cohorts [256]. Taken together, AI-assisted multi-omics data analysis can serve as a powerful tool for evaluating the level of immune cell infiltration in TME and predicting the efficacy of immunotherapy, helping clinicians understand the immune status of patients and selecting candidates with a higher rate of response and a better prognosis for ICI therapy.

In addition to the integration of multi-omics data, the burgeoning single-cell transcriptomic and spatial transcriptomic technologies are driving the application of ML in the prediction of breast cancer treatment response. Hu et al. revealed the heterogeneity and developmental dynamics of infiltrating B-cell subgroups in TNBC by single-cell transcriptional profiling and found that B-cell signaling was significantly associated with improved survival in patients [257]. Wu et al. mapped the comprehensive spatial transcriptional profile of breast cancer cellular structures and used cellular indexing of transcriptomes and epitopes by sequencing for immunophenotyping, providing high-resolution immune profiles [258]. To characterize the relationship between tissue structure, its therapeutic dynamics and immunotherapy response in TNBC, Wang et al. used imaging mass cytometry^3^ to accurately quantify the phenotype, activation status and spatial location of tumor cells sampled at three timepoints from patients enrolled in the NeoTRIP randomized trial of neoadjuvant ICI. Researchers found that combining tissue features before and during treatment could best predict ICI response [259]. Advances in single-cell and spatial transcriptomics technologies have revealed complex interactions between cells and genes, supplying detailed bioinformatics data for ML models. ML models can perform network analysis based on this information to gain a deeper understanding of the dynamic behaviors of biological systems, capture more accurately the types and states of cells as well as the subtle changes in tissue structure, and mine out key immunotherapeutic biomarkers and gene expression patterns.

Although the majority of studies have proven that their models performed as well as or better than physicians, there are only a handful of successful real-world applications [260]. Lack of unified databases, industry standards, specialized clinical application situations, and policy and regulatory assistance may lead to practical challenges. Therefore, we should promote cross-domain code sharing and the development of standardized datasets, encourage multidisciplinary and multi-center collaboration, establish a strong regulatory system. Additionally, data privacy and security concerns must be addressed to ensure that patients’ sensitive medical information is protected [261].

## Conclusions

While immunotherapy has made remarkable achievement in treating various cancers, its application in breast cancer has presented both opportunities and challenges. For high-risk eTNBC and PD-L1+ mTNBC, pembrolizumab combined with chemotherapy has emerged as part of the current standard of care. Meanwhile, real-world data from these regimens will provide a better understanding of the risks and benefits. Among the combination regimens of ICIs and various agents, ICIs coupled with antibody-drug conjugates (ADCs) appears most promising in all breast cancer subtypes. Extensive research is underway to explore the immune landscape of HR+ and HER2+ disease, where ICIs might replicate the survival benefits observed in TNBC. Despite these advancements, several critical issues remain including the optimal partners for ICIs, the appropriate duration of ICI therapy for early-stage disease and the treatment of patients in the metastatic setting after receiving ICI. Future research efforts ought to focus on establishing predictive models based on a panel of biomarkers and identifying optimal benefit subpopulations for immunotherapy. Special attention should be paid to recognizing and mitigating the additional toxicity of these combinations while maintaining a favorable risk-benefit ratio. Rapidly evolving AI technologies has greatly facilitated the development of refined subtype classification and precision immunotherapy for breast cancer, contributing to reshaping the future landscape of breast cancer immunotherapy.

## Data Availability

No datasets were generated or analysed during the current study.

## Abbreviations

**AC**: Doxorubicin/cyclophosphamide
ACTs: Adoptive cell therapies
ADCC: Antibody-dependent cell-mediated cytotoxicity
ADCs: Antibody–drug conjugates
AEs: Adverse events
AI: Artificial intelligence
AI-CDSS: Artificial intelligence-powered clinical decision support systems
AIDD: Artificial intelligence-driven drug design
APC: Antigen-presenting cell
Amp: Amplification
Atezo: Atezolizumab
B2M: β2-Microglobulin
BiTE: Bispecific T-cell engager
BLIS: Basal-like immune-suppressed
BM: Basal/mesenchymal-like
BsAbs: Bispecific antibodies
CAR T cell: Chimeric antigen receptor T cell
CARs: Chimeric antigen receptors
Camre: Camrelizumab
CBR: Clinical benefit rate
CD8 +: CD8-positive
CDK4/6: Cyclin-dependent kinase 4/6 inhibitor
CI: Confidence interval
CLA: Classical
CPS: Combined positive score
CR: Complete response
CTAs: Cancer-testis antigens
CTC: Circulating tumor cells
ctDNA: Circulating tumor DNA
CTLA-4: Cytotoxic T-lymphocyte–associated protein 4
Dato-DXd: Datopotamab deruxtecan
DCR: Disease control rate
DCs: Dendritic cells
ddAC/EC: Dose dense anthracycline/epirubicin and cyclophosphamide
DDFS: Distant disease-free survival
DFS: Disease-free survival
dMMR: Deficiency of MMR
DoR: Duration of response
Durva: Durvalumab
EC: Epirubicin/cyclophosphamide
ECM: Extracellular matrix
EFS: Event-free survival
EMT: Epithelial-mesenchymal transition
ER + /HER2-: Estrogen receptor-positive/HER2-negative
eTNBC: Early triple-negative breast cancer
FDA: Food and Drug Administration
FRα: Folate receptor alpha
FUL: Fulvestrant
GEP: Gene expression profile
GZMB: Granzyme B
HER2: Human epidermal growth factor receptor 2
HER2-: Human epidermal growth factor receptor 2-negative
HLA: Human leukocyte antigen
HP: Trastuzumab-pertuzumab
HR: Hormone receptor
HR + /HER2-: Hormone receptor-positive/HER2-negative
ICIs: Immune checkpoint inhibitors
ICs: Immune cells
iDFS: Invasive disease-free survival
IDO1: Indoleamine2,3-dioxygenase1
IFN: Interferon
IHC: Immunohistochemistry
irAEs: Immune-related adverse events
ITT: Intention-to-treat
LAG-3: Lymphocyte-activation gene 3
LAR: Luminal androgen receptor
LDH: Lactate dehydrogenase
LET: Letrozole
LUM: Luminal-like
LN: Lymph node
mAb: Monoclonal antibody
MAGE-12: Melanoma antigen gene protein-12
MBC: Metastatic breast cancer
MEK: Mitogen-activated extracellular signal-regulated kinase
MES: Mesenchymal-like
MHC: Major histocompatibility complex
ML: Machine learning
MMR: Mismatch repair
MSI: Microsatellite instability
MSI-H: Microsatellite instability-high
Mut: Mutation
mTNBC: Metastatic triple-negative breast cancer
MUC1: Mucin 1
mut/Mb: Mutations/megabase
MWA: Microwave ablation
Mφ: Macrophage
NACT: Neoadjuvant chemotherapy
Nivo: Nivolumab
NK: Natural killer
NMPA: National Medical Products Administration
NVB: Vinorelbine
NYESO-1: New York esophageal squamous cell carcinoma 1
OR: Odds ratio
ORR: Objective response rate
OS: Overall survival
PARP: Poly (ADP-ribose) polymerase
pCR: Pathologic complete response
PD-1: Programmed cell death 1
PD-L1-: PD-L1-negative
PD-L1: Programmed death ligand 1
PD-L1 +: PD-L1-positive
Pembro: Pembrolizumab
PFN: Perforin
PFS: Progression-free survival
pMMR: Proficiency of mismatch repair
PR: Partial response
REF: Reference
RTK: Receptor tyrosine kinase
sCD163: Soluble variant of CD163
SCNAs: Somatic copy number alteration
SD: Stable disease
SG: Sacituzumab govitecan
Sparta: Spartalizumab
RTK: Receptor tyrosine kinase
TAAs: Tumor-associated antigens
TAC: Tumor-associated carbohydrate
TCR: T cell receptor
tBRCAm: Tumor BRCA mutations
TCR: T cell receptor
T-DM1: Trastuzumab emtansine
T-DXd: Trastuzumab deruxtecan
THP: Taxane plus trastuzumab and pertuzumab
TIGIT: T cell immunoglobulin and ITIM domain
TILs: Tumor-infiltrating lymphocytes
TLS: Tertiary lymphoid structures
TIM-3: T cell immunoglobulin and mucin domain-3 protein
TMB-H: High TMB
TME: Tumor microenvironment
TNBC: Triple-negative breast cancer
TNF: Tumour necrosis factor
TRAEs: Treatment-related adverse events
TRM: Tissue-resident memory
VEGF: Vascular endothelial growth factor
wP: Weekly paclitaxel
WT1: Wilms' tumor protein
